# Antitumor activity of AZD0754, a dnTGF**β**RII-armored, STEAP2-targeted CAR-T cell therapy, in prostate cancer

**DOI:** 10.1172/JCI169655

**Published:** 2023-11-15

**Authors:** Peter Zanvit, Dewald van Dyk, Christine Fazenbaker, Kelly McGlinchey, Weichuan Luo, Jessica M. Pezold, John Meekin, Chien-ying Chang, Rosa A. Carrasco, Shannon Breen, Crystal Sao-Fong Cheung, Ariel Endlich-Frazier, Benjamin Clark, Nina J. Chu, Alessio Vantellini, Philip L. Martin, Clare E. Hoover, Kenesha Riley, Steve M. Sweet, David Chain, Yeoun Jin Kim, Eric Tu, Nathalie Harder, Sandrina Phipps, Melissa Damschroder, Ryan N. Gilbreth, Mark Cobbold, Gordon Moody, Emily E. Bosco

**Affiliations:** 1 Early Oncology Research,; 2 Biologics Engineering,; 3 Discovery Sciences, Oncology R&D, and; 4 Oncology Translational Medicine, Oncology R&D, AstraZeneca, Gaithersburg, Maryland, USA.; 5 Clinical Pathology Patient Safety, BioPharmaceuticals R&D, AstraZeneca, Cambridge, United Kingdom.; 6 Computational Pathology, AstraZeneca, Munich, Germany.

**Keywords:** Oncology, Prostate cancer

## Abstract

Prostate cancer is generally considered an immunologically “cold” tumor type that is insensitive to immunotherapy. Targeting surface antigens on tumors through cellular therapy can induce a potent antitumor immune response to “heat up” the tumor microenvironment. However, many antigens expressed on prostate tumor cells are also found on normal tissues, potentially causing on-target, off-tumor toxicities and a suboptimal therapeutic index. Our studies revealed that six-transmembrane epithelial antigen of prostate-2 (STEAP2) was a prevalent prostate cancer antigen that displayed high, homogeneous cell surface expression across all stages of disease with limited distal normal tissue expression, making it ideal for therapeutic targeting. A multifaceted lead generation approach enabled development of an armored STEAP2 chimeric antigen receptor T cell (CAR-T) therapeutic candidate, AZD0754. This CAR-T product was armored with a dominant-negative TGF-β type II receptor, bolstering its activity in the TGF-β–rich immunosuppressive environment of prostate cancer. AZD0754 demonstrated potent and specific cytotoxicity against antigen-expressing cells in vitro despite TGF-β–rich conditions. Further, AZD0754 enforced robust, dose-dependent in vivo efficacy in STEAP2-expressing cancer cell line–derived and patient-derived xenograft mouse models, and exhibited encouraging preclinical safety. Together, these data underscore the therapeutic tractability of STEAP2 in prostate cancer as well as build confidence in the specificity, potency, and tolerability of this potentially first-in-class CAR-T therapy.

## Graphical abstract

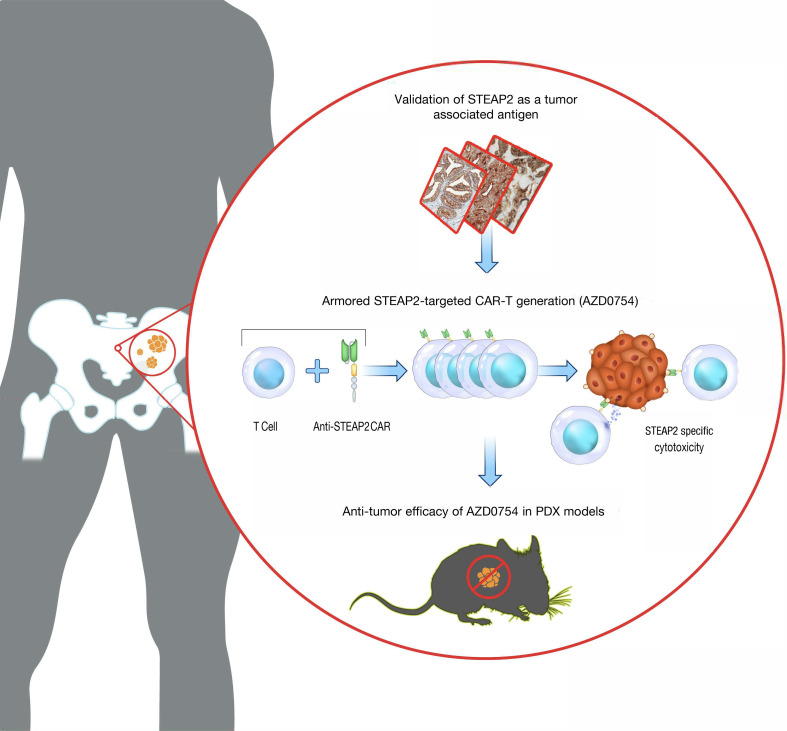

## Introduction

Prostate cancer is the most common non-skin cancer diagnosed in men and has the second-highest mortality rate of any cancer (1). Despite advancements in biomarkers and therapeutic approaches, the 5-year relative survival rate of patients with distant disease remains only 30% (2). Advanced disease often metastasizes to bone and becomes refractory to therapy, highlighting the urgent need for innovative therapeutic options for these patients.

Prostate cancer is generally considered an immunologically “cold” tumor type that is insensitive to immunotherapy. This can be due to multiple factors, including the immunosuppressive transforming growth factor-β–rich (TGF-β–rich) tumor microenvironment (TME) of prostate cancer, dysfunctional T cells, tumor loss of major histocompatibility complex (MHC) class I, and a dearth of tumor-associated antigens (3, 4). To circumvent these factors, targeting surface antigens on tumors through cellular therapy can serve to induce a potent antitumor immune response to “heat up” the TME. Many antigens expressed on prostate tumors are also found on critical normal tissues, potentially causing on-target, off-tumor toxicities and a suboptimal therapeutic index. The identification of suitable prostate tumor antigens has been a persistent challenge in the field.

Large-scale genomic and proteomic efforts have identified six-transmembrane epithelial antigen of prostate-2 (STEAP2; also known as STAMP1) as a superior prostate antigen for therapeutic targeting (5, 6). Consistent with this discovery, it has been shown that STEAP2 expression is abundant across all stages of prostate cancer and can be used as a prognostic biomarker owing to its correlation with Gleason score (7–9). Although STEAP2 gene expression and protein detection have been established by IHC, the literature on subcellular localization of the protein remains inconsistent. This is probably due to a lack of specificity of commercial reagents and the challenging protein structure of STEAP2, which complicate efforts to generate an antibody targeted to the extracellular domain (ECD). Our antibody discovery campaign identified a unique anti-STEAP2 monoclonal antibody that was instrumental in characterizing the highly homogeneous STEAP2 protein expression in tumors and the limited normal tissue expression profile that would be amenable to therapeutic intervention.

Although chimeric antigen receptor T cell (CAR-T) therapies have revolutionized medicine and improved the lives of many cancer patients, trials in solid tumors have yet to replicate the high response rates observed with CAR-T products in hematological malignancies. Nevertheless, prostate cancer has been one of the most widely investigated solid tumor types for CAR-T therapies, as clinical-stage cell products have most often targeted prostate-specific membrane antigen (PSMA) and prostate stem cell antigen (PSCA). Although these early-stage clinical trials have demonstrated signs of antitumor activity (as measured by prostate-specific antigen declines and radiographic improvement in advanced disease), both antigens have known normal tissue expression liabilities that may ultimately serve to narrow the therapeutic index of these therapies (10).

Considerable effort has been invested in understanding the immunosuppressive forces that limit the infiltration, expansion, and effector function of CAR-Ts in the prostate cancer TME. Genomic analyses from 2 large-scale studies on clinical gene expression have demonstrated elevation of TGF-β1 gene expression in prostate cancer, even in metastatic lesions (11, 12). Consistent with these findings, recent reports have revealed that bone metastases secondary to prostate cancer promote osteoclast-mediated bone resorption, which releases high levels of TGF-β. This TGF-β can confer resistance to immunotherapy and inhibit T cell antitumor activity (13).

Considering the immunosuppressive nature of TGF-β, we undertook a series of studies to investigate the feasibility of targeting the STEAP2 prostate antigen with CAR-Ts that were armored to withstand the TGF-β–rich immunosuppressive environment found in prostate tumors. The nonclinical studies described herein led to the generation of a specific STEAP2-targeted CAR-T product that exhibits persistence and antitumor activity despite TGF-β–mediated suppression. These data reveal that human-murine cross-reactive STEAP2 CAR-Ts can effectively elicit antitumor activity without toxicity in in vivo models of prostate cancer, including subcutaneous cell line xenografts, orthotopic models of bone metastases, and patient-derived xenograft models. The results provide evidence that the dnTGFβRII-armored STEAP2-targeted CAR-T product, AZD0754, may present a therapeutic option for metastatic castration-resistant prostate cancer.

## Results

**Figure 1:**
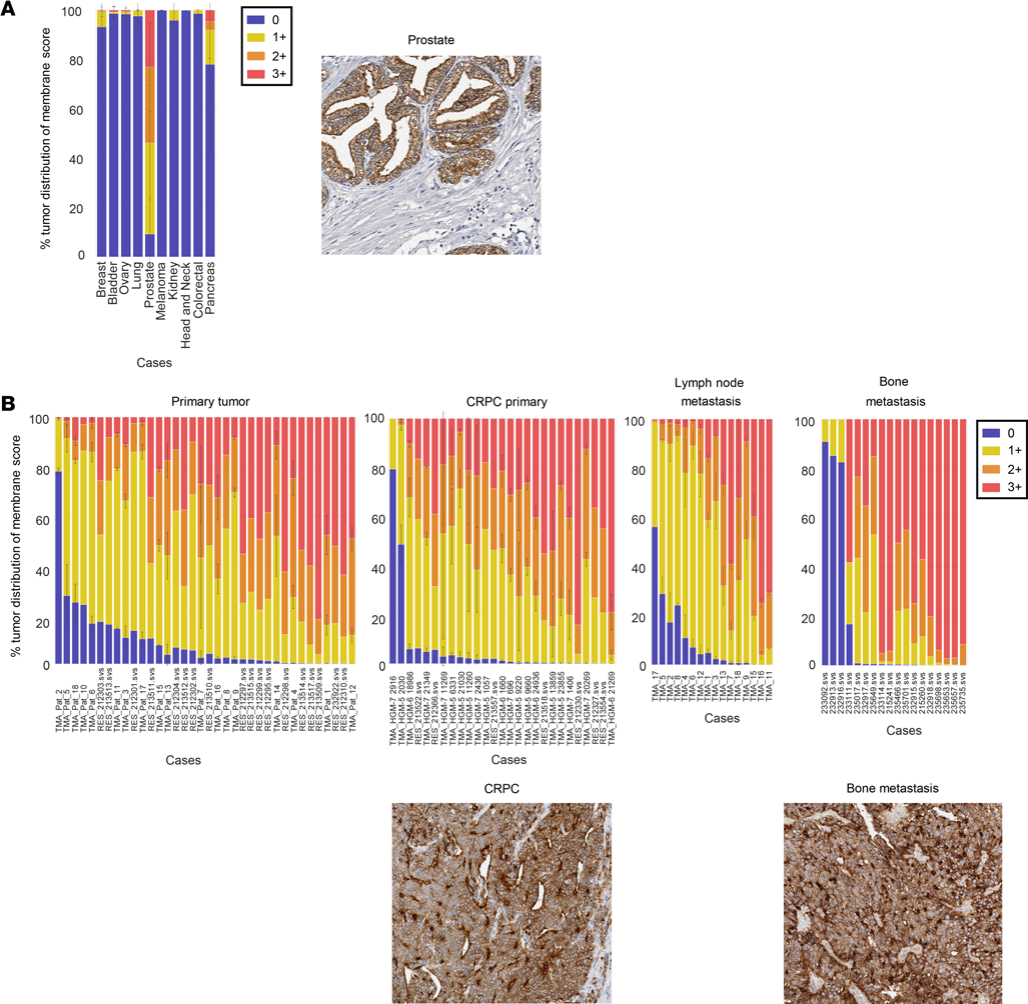
Prevalence of STEAP2, a prostate tumor–associated antigen, throughout disease progression. (**A**) IHC studies of a broad TMA demonstrated membrane STEAP2 expression exclusively in prostate cancer. An example of STEAP2 IHC staining in normal prostate tissue is shown. (**B**) As in **A**, TMAs containing primary prostate cancer, CRPC, and prostate lymph node metastases, as well as decalcified full-face sections of prostate cancer bone metastases, were evaluated by IHC for STEAP2 membrane expression. Representative images of STEAP2 in CRPC and bone metastases are shown under the IHC graphs. All IHC images shown are at ×20 original magnification.

To interrogate the rationale for targeting STEAP2, we first aimed to understand the expression profile and localization of the target. We confirmed literature reports of STEAP2 overexpression in prostate cancer by using large-scale genomics screening in the QIAGEN Omicssoft OncoLand database, where prostate adenocarcinoma and normal prostate tissue displayed substantially higher gene expression levels than all other tumor types and normal tissues profiled (Supplemental Figure 1; supplemental material available online with this article; https://doi.org/10.1172/JCI169655DS1). Next, we developed a STEAP2-specific rabbit polyclonal IHC reagent to evaluate the prevalence and localization of protein expression in broad tissue microarrays (TMAs), where high, homogeneous expression levels were found to be restricted to prostate tumors (Figure 1A and Supplemental Figure 2). When IHC was used to profile TMAs from various stages of prostate cancer progression, including primary, castration-resistant prostate cancer (CRPC), lymph node metastases, and decalcified full-face sections from bone metastases, more than 85% of all tumor samples throughout all disease progression stages showed more than 75% of all tumor cells as positive for cell surface STEAP2. Representative images of CRPC and bone metastatic samples showed predominantly circumferential and apical staining patterns (Figure 1B). Finally, to investigate the expression pattern of STEAP2 in healthy human tissue, ISH staining was conducted concomitantly with IHC to confirm protein abundance and localization, using a STEAP2-specific probe in a normal tissue TMA (Table 1). Although low to moderate STEAP2 gene expression was identified by ISH in several normal human tissues, the signal was not shown as cell surface protein expression as identified by IHC. As an orthogonal, antibody-independent approach to assess STEAP2 protein expression, laser capture microdissection coupled with targeted proteomics was used in regions of interest. Consistent with the IHC and ISH data, targeted proteomic analysis of STEAP2 revealed high levels (median concentration, >500 amol/μg) in prostate tissue and low levels (median concentration, <50 amol/μg, below the 46 amol/μg lower limit of quantification for the assay) in non-prostate tissues (Table 1). Only normal prostate was found to have high, homogeneous STEAP2 expression across all healthy tissues profiled.

**Figure 2:**
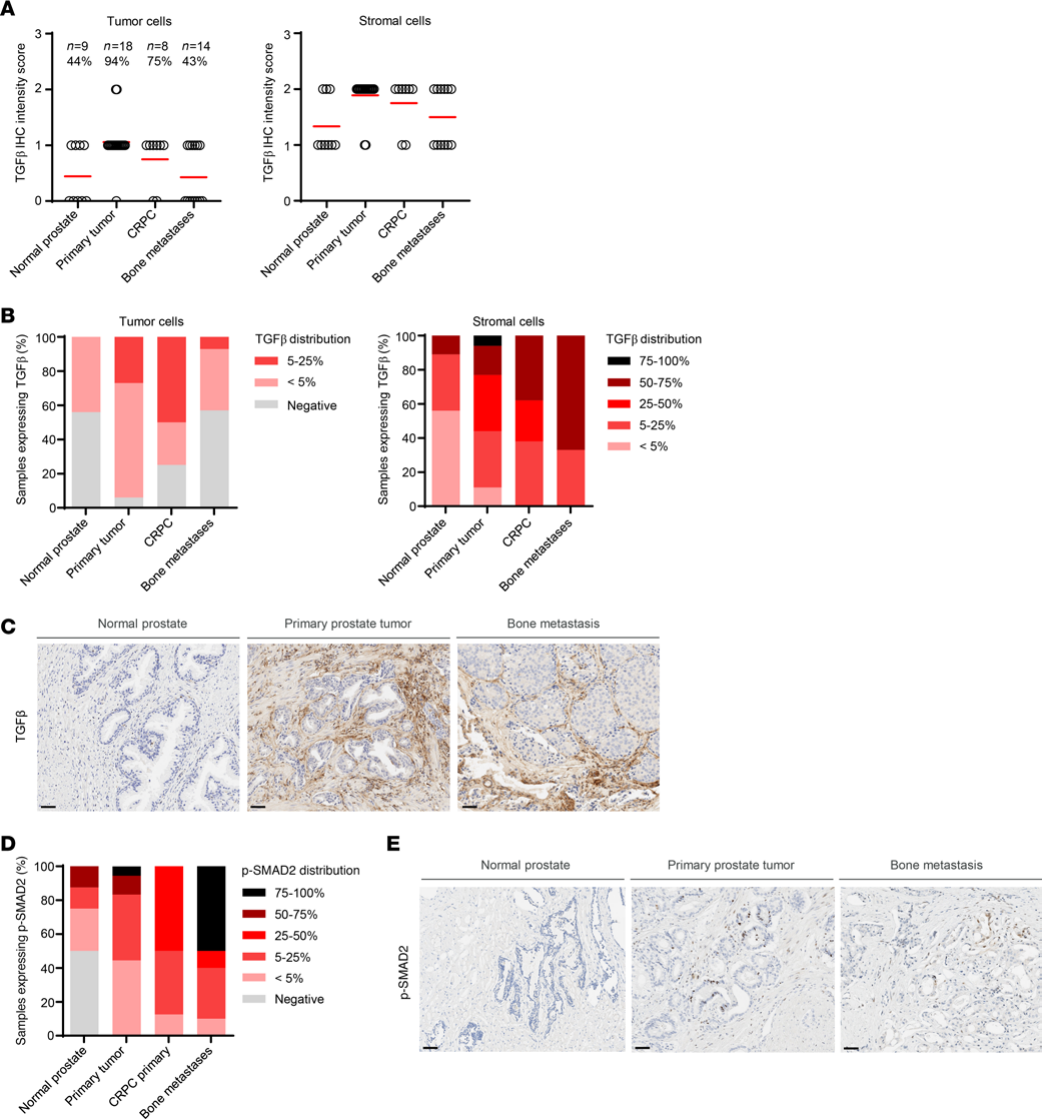
Prevalence of TGF-β throughout prostate cancer progression. (**A**) The same prostate cancer TMA samples shown in Figure 1 were profiled for expression of TGF-β by IHC. The TGF-β staining intensity in tumor and stromal cells was quantified. (**B**) Distribution of TGF-β staining in tumor and stromal cells. (**C**) Representative images of TGF-β staining in normal prostate sample, primary prostate tumor, and prostate cancer bone metastatic sample, highlighting the contribution of tumor and stromal cell TGF-β in the TME. (**D**) IHC for p-SMAD2 was performed on the samples shown in **A**, and the distribution of expression in each prostate cancer disease subset was quantified. (**E**) Representative images of p-SMAD2 staining quantified in **D**. All images include a 50 μm scale bar at bottom left.

To understand the level of TGF-β present throughout prostate cancer progression, the same tumor samples shown in Figure 1B were stained by IHC for TGF-β. The tumor cells mostly displayed a TGF-β intensity of 0 to 1+, whereas the intensity was increased to 1 to 2+ in the tumor stromal compartment in samples across the stages of disease progression (Figure 2A). Similarly, the distribution of the stain throughout each tumor section was relatively low and varied across the tumors, but distribution increased with disease progression in the stromal cells (Figure 2B). Representative IHC images of TGF-β staining in a normal prostate, primary prostate cancer, and a prostate cancer bone metastasis are shown in Figure 2C highlighting the high levels of TGF-β staining in tumor stroma and immune cells compared with normal prostate. It is likely that this TGF-β is in the active form, because the distribution of phosphorylated SMAD2 (p-SMAD2), a downstream component in the TGF-β signaling axis, was similarly increased with advanced disease (Figure 2, D and E).

The STEAP2 protein has been largely unstudied because its multiple transmembrane domains pose a marked challenge to antibody development. The lack of STEAP2-specific commercial antibodies is due to the limited length of STEAP2 extracellular loops and their near-total conservation across species and high homology with other STEAP family members. A recent publication reported the development of a STEAP2 functional antibody that demonstrated binding in a cholesterol-dependent fashion (9). The unstable epitope of the antibody in that study, however, poses challenges to its use in a therapeutic setting.

**Figure 3:**
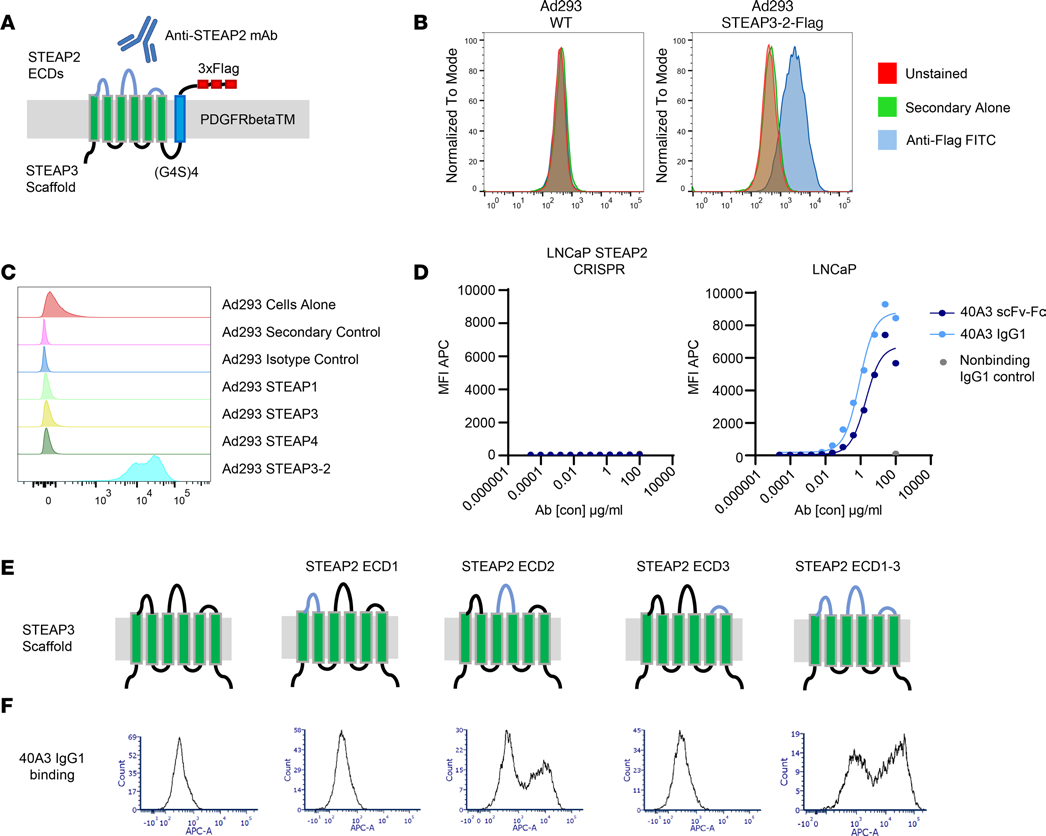
Generation of an anti-STEAP2 antibody that demonstrates specificity in model systems. (**A**) Chimera containing the STEAP3-GS-PDGFRβTM-FLAG amino acid sequence backbone with the sequence of the STEAP2 extracellular loops grafted on to establish cell surface localization. (**B**) FACS analysis demonstrating robust and stable expression of the STEAP3-2–FLAG chimera and, by extrapolation, the STEAP3-2 (non-tagged) used for antibody generation, at the cell surface of Ad293 cells. (**C**) 40A3 scFv-Fc was tested for binding to Ad293 cells expressing human STEAP family members (STEAP1, 2, 3, and 4). (**D**) Multiple scFv-Fcs and full-length IgG1 antibodies were screened for binding to antigen-positive (Ad293 STEAP3-2, Ad293 STEAP3-2 murine, and LNCaP) cells and antigen-negative (Ad293 and LNCaP STEAP2 CRISPR) cell lines. Representative FACS titration graphs show binding curves for the 40A3 scFv-Fc, 40A3 IgG1, and nonbinding IgG1 as a negative control in the LNCaP STEAP2 CRISPR and LNCaP cell lines. Anti–human Fc secondary antibodies conjugated with Alexa Fluor 647 were used for detection of scFv-Fc or IgG1 binding to cells by flow cytometry. (**E**) Models of STEAP3-STEAP2 ECD chimeras used to assess 40A3 IgG domain recognition. (**F**) FACS analysis showing preferential binding of 40A3 IgG to ECD2 of STEAP2 on Ad293 cells expressing STEAP3-2 chimeras. All FACS results are representative of *n* > 3 experimental replicates.

To generate STEAP2-specific antibodies to stable epitopes, we used 2 strategies. First, we used a cell immunization hybridoma campaign that allowed the murine immune system to recognize the STEAP2 protein in its native conformation. In previous research, we were not able to drive expression and cell surface localization of STEAP2 in a non-prostate cell setting, such as in Ad293 cells (data not shown). This complicating factor for the study of STEAP2 biology was also reported by others (9) and could suggest a potential requirement for prostate-specific chaperones or cell membrane scaffold proteins to enforce STEAP2 cell surface expression. To circumvent this problem, we created a chimeric cell line named STEAP3-2, in which we grafted the STEAP2 extracellular loops onto the backbone of the STEAP3 protein to exploit the cell surface localization of STEAP3 (Supplemental Figure 4). To enable detection of the chimeric protein on the cell surface, platelet-derived growth factor receptor β (PDGFRβ) transmembrane domain with a triple FLAG tag was fused to the intracellular STEAP3-2 C-terminus (Figure 3A). Cell surface localization of STEAP3-2–FLAG was subsequently confirmed by flow cytometry, using anti-FLAG for detection (Figure 3B). Humanized Del-1 mice were immunized with Ad293 STEAP3-2 (non-tagged) chimeric cells for lead generation. Second, we undertook a complex B cell hybridoma enrichment screening approach to discover functional STEAP2-targeting antibodies (Supplemental Figure 3). In short, after murine immunization, we harvested spleens and lymph nodes for B cells and enriched for STEAP2-specific B cell clones through a deselection step with an LNCaP STEAP2 CRISPR knockout engineered cell line. This allowed all remaining nonbinding clones to proceed to hybridoma fusion and scale-up. The conditioned hybridoma supernatants were subjected to primary specificity screening on the LNCaP and LNCaP STEAP2 CRISPR isogenic cell line pair, as well as the Ad293 and Ad293 STEAP3-2 cells.

The secondary screening entailed elimination of clones that displayed binding to Ad293 cell lines overexpressing STEAP1, 3, or 4 family members and selection of those that exhibited binding to cells overexpressing murine STEAP2 ECD loops, Ad293 STEAP3-2 murine cells. The V genes of these resulting STEAP2-specific, murine cross-reactive hybridomas were recovered for monoclonal IgG production, and selective binding was again validated with similar criteria. IgG clones were converted to scFv-Fc, and in vitro binding assays were performed by FACS to evaluate the specificity of 40A3 scFv-Fc for STEAP2 and lack of background binding to antigen-negative cell lines and other STEAP family members (Figure 3C). Next, we assessed on-cell binding affinity with antigen-positive and -negative cell lines to quantify the relative affinities of 40A3 scFv for human and murine STEAP2 as 20.2 nM and 28.2 nM, respectively (Figure 3D and Table 2). Chimeric cell lines were generated to pinpoint STEAP2 ECD recognition by the lead antibody, 40A3 IgG, as a surrogate for the CAR scFv. To this end, STEAP3 was again used as a scaffold to present STEAP2 ECDs individually or in combination (Figure 3E). The 40A3 IgG showed a distinct preference by FACS for STEAP2 ECD2, the longest of the 3 extracellular loops (Figure 3F). The involvement of STEAP2 ECDs 1 and 3 cannot be excluded, but the data strongly suggest that the major epitope for 40A3 IgG recognition resides within ECD2.

Lead scFvs were cloned into a second-generation CAR construct with 4-1BB and CD3ζ signaling domains. More specifically, the CAR genes were constructed in a pESRC lentivirus construct by fusing of the following in series: human CSFR2 signal peptide, scFv, human IgG4 hinge with S228P mutation, human CD28 transmembrane domain, human 4-1BB cytoplasmic domain, and human CD3ζ cytoplasmic domain. Upon transduction into human T cells, these CAR-T clones were further triaged for specificity of killing on antigen-positive and -negative cell lines to yield 40A3Bz as the lead molecule.

**Figure 4:**
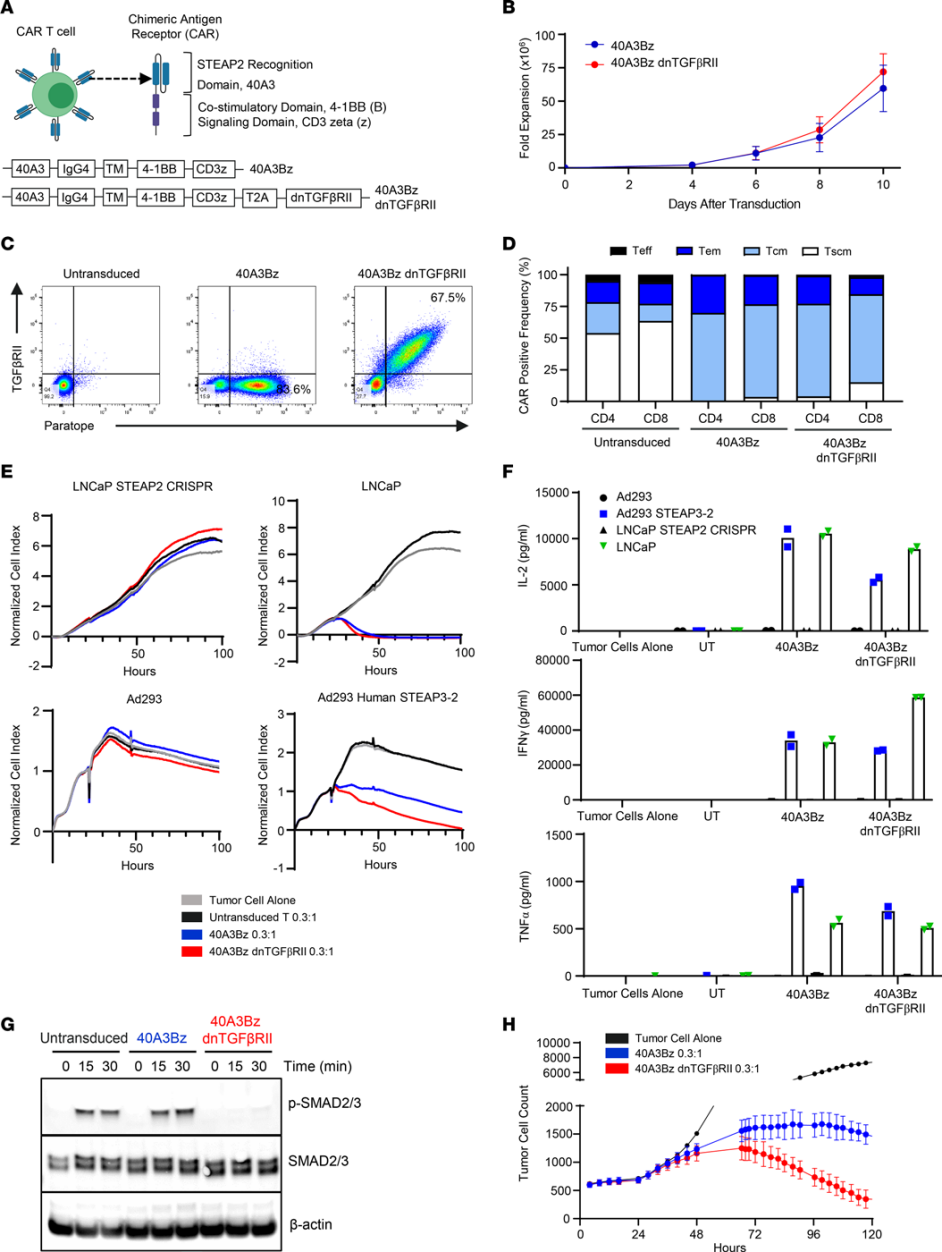
In vitro cytolytic activity of 40A3Bz dnTGFβRII CAR-Ts. (**A**) Components of the STEAP2 unarmored and armored lentivirus constructs used in generation of the CAR-Ts. (**B**) Viable cell expansion of CAR-Ts was assessed for 10 days after lentivirus transduction in *n* = 3 donors. Data represent mean ± SEM. (**C**) The 40A3Bz cells and 40A3Bz dnTGFβRII STEAP2 CAR-Ts were evaluated by flow cytometry at day 9 after transduction to assess CAR positivity and cell surface expression of dnTGFβRII, compared with untransduced T cells from the same donor. (**D**) CAR-Ts from **C** were stained for phenotypic surface markers including CD45RO and CD62L and analyzed by flow cytometry. Naive (CD45RO^–^CD62L^+^), central memory (CD45RO^+^CD62L^+^), effector memory (CD45RO^+^CD62L^–^), and effector (CD45RO^–^CD62L^–^) T cells were used. (**E**) CAR-Ts from **C** were cocultured with antigen-positive (Ad293 STEAP3-2 and LNCaP) and antigen-negative (Ad293 and LNCaP STEAP2 CRISPR) cell lines. Killing of target cells was measured over 100 hours with the xCELLigence impedance assay. Data are an average of duplicate. (**F**) Supernatants from the same coculture experiments were collected 24 hours after addition of CAR-Ts, and cytokines (IFN-γ, TNF-α, and IL-2) were measured by Meso Scale Discovery (MSD) electrochemiluminescence assay. Data are an average of duplicate. (**G**) STEAP2 CAR-Ts were subjected to FACS for CAR positivity and starved overnight before stimulation with recombinant human TGF-β treatment. Cell lysates were generated during the indicated time course, and Western blotting was performed to evaluate levels of p-SMAD2/3, total SMAD2/3, and β-actin in each sample. (**H**) STEAP2 CAR-Ts were cocultured with antigen-positive cells (C4-2 cells stably expressing mKate2 red fluorescent protein) at a 0.3:1 ratio in the presence of 30 ng/mL recombinant TGF-β. C4-2 cell viability was monitored over 120 hours with the Incucyte live cell analysis system (Sartorius) in triplicate. Data represent mean ± SEM. Data in **B**–**F** and **H** are representative of the results obtained in 3 or more independent experiments with CAR-Ts prepared from 3 healthy donors, and **G** was performed twice with 2 donors.

The 40A3 scFv was selected as a lead in the aforementioned CAR format using an EF1-α promoter and 4-1BB and CD3ζ signaling domains, as well as in an armored format containing the dnTGFβRII that has been tested clinically (14, 15) (Figure 4A). This promoter and CAR endodomain were selected based on prior in-house experience with other CAR constructs as well as clinical experience with tisagenlecleucel and lisocabtagene maraleucel. Data from the literature suggest that the inclusion of a 4-1BB rather than a CD28 endodomain may favor the outgrowth of younger central memory cells with improved persistence while mitigating excessive immune activation leading to cytokine release syndrome (CRS) and CAR-T–related neurotoxicity (16). Expansion of the CAR-Ts from 3 donors was monitored over 10 days after transduction, and a similar degree of fold expansion of the 40A3Bz cells and 40A3Bz dnTGFβRII CAR-Ts was observed (Figure 4B). Phenotypic characterization was performed on 40A3Bz cells and 40A3Bz dnTGFβRII CAR-Ts that had been expanded for 10 days. In an example from a representative donor, a total of 84% CAR-positive T cells were identified by flow cytometry using a monoclonal anti-40A3 paratope antibody to assess expression in the 40A3Bz cells, whereas 68% of the 40A3Bz dnTGFβRII-transduced T cells revealed dual positivity for binding to the paratope antibody and the TGFβRII antibody (Figure 4C). CAR-positive T cells from all conditions had a predominantly naive/central memory phenotype at the end of the expansion process, as determined by staining with CD62L and CD45RO antibodies, where effector T cells were CD62L^–^CD45RO^–^, effector memory T cells were CD62L^–^CD45RO^+^, central memory T cells were CD62L^+^CD45RO^+^, and memory stem T cells were CD62L^+^CD45RO^–^ (Figure 4D). We also confirmed that CAR-Ts had low levels of differentiation and exhaustion markers (CD45RA, CD69, KLRG1, CD127, PD-1, LAG3; data not shown). The 40A3Bz dnTGFβRII CAR-Ts that had been expanded for 10 days were shown to kill antigen-positive target cells (LNCaP or Ad293 STEAP3-2) in a fashion similar to the killing of unarmored STEAP2 CAR-Ts when placed in coculture at an effector-to-target (E/T) ratio of 0.3:1 by xCELLigence real-time cytotoxicity assay (Figure 4E). In addition, 40A3Bz dnTGFβRII STEAP2 CAR-Ts displayed multifunctionality by releasing proinflammatory cytokines after 24 hours of coculture (Figure 4F). Importantly, no cytotoxic activity or release of proinflammatory cytokines was observed following coculture of armored STEAP2 CAR-Ts with antigen-negative targets (LNCaP STEAP2 CRISPR, Ad293), indicating that T cell activity was STEAP2 antigen dependent with no evidence of tonic CAR-T signaling.

To further delineate the mechanism of target-dependent specificity of the 40A3Bz dnTGFβRII CAR-Ts in the coculture cytotoxicity assays, a 40A3 blocking antibody or isotype control antibody was titrated into the coculture at 0.2, 2, 20, or 200 μg/mL, and CAR-T killing of LNCaP and LNCaP STEAP2 CRISPR cell lines was analyzed (Supplemental Figure 5A). The 40A3 blocking antibody clearly inhibited CAR-T cytotoxicity in a dose-dependent manner at 200 and 20 μg/mL, whereas the isotype control had no impact on CAR-T killing. This was also evident in the amount of IFN-γ detected in the supernatant at 24 hours. As expected, no tumor cell killing or cytokine production was detected in the LNCaP STEAP2 CRISPR–negative control line (Supplemental Figure 5B).

In order to determine the receptor density necessary to elicit CAR-T cytolytic activity, quantitative anti-STEAP2 FACS was performed on a titration range of cell lines suspected of STEAP2 surface expression based on gene expression profiles. STEAP2 antigen binding capacity was found to correlate with IHC scoring on the cell line pellets (Supplemental Figure 6A). Accordingly, these cell lines were subjected to coculture with 40A3Bz dnTGFβRII CAR-Ts at an E/T ratio of 1:1. Supernatants were collected after 24 hours and evaluated for IFN-γ cytokine production by Meso Scale Discovery assay. As expected, IFN-γ levels correlated with cell surface expression levels of STEAP2 (Supplemental Figure 6B). Identical analyses were performed for a variety of primary, immortalized, and cancer cell lines to search for potential CAR-T activation by cells that could exhibit sub-detectable levels of STEAP2 by FACS, but no appreciable cytokine release was detected in cocultures with cells other than the positive control C4-2, LNCaP, and Ad293 STEAP3-2 cells (Supplemental Figure 7).

Next, to provide functional validation of dnTGFβRII armoring, we purified >97% CAR-positive 40A3Bz cells and 40A3Bz dnTGFβRII CAR-Ts through FACS at day 4 after transduction. These cells were expanded to day 15 and starved in X-VIVO 15 medium (Lonza Bioscience) for 17 hours before stimulation with 1 ng/mL recombinant human TGF-β. Signal transduction downstream of the native TGFβRII was compared between the armored and unarmored CAR-Ts. Western blots for p-SMAD2/3, total SMAD2/3, and β-actin confirmed a substantial abrogation of TGF-β–mediated signaling in the 40A3 dnTGFβRII CAR-Ts compared with 40A3Bz CAR or untransduced cells alone (Figure 4G). To evaluate cytotoxic activity in the presence of TGF-β–mediated immunosuppression, using levels estimated from the prostate cancer bone TME (13), 40A3Bz cells and 40A3Bz dnTGFβRII CAR-Ts were cocultured with STEAP2-expressing C4-2 cells in the presence of 30 ng/mL TGF-β. Unlike 40A3Bz CAR-Ts, 40A3Bz dnTGFβRII cells maintained their cytotoxic activity despite the presence of high levels of TGF-β in the medium, confirming that TGF-β pathway signaling was disrupted in these cells (Figure 4H).

Because 40A3Bz CAR-Ts are cross-reactive with murine STEAP2, we next questioned the in vivo tolerability and antitumor efficacy of STEAP2 targeting. NSG mice were implanted with C4-2 xenografts and randomized when tumors reached approximately 175 mm^3^ before being treated with 3 × 10^6^, 7 × 10^6^, or 21 × 10^6^ 40A3Bz CAR-positive cells per mouse. Six mice from each group were sacrificed at days 10 and 20 after CAR-T infusion, and normal tissues of heart, lung, liver, kidney, spleen, prostate, and skin were analyzed for signs of CD3 infiltration by IHC and tissue damage by H&E. Despite considerable tumor growth inhibition in all CAR-T treatment groups throughout the study (Supplemental Figure 8A), there appeared to be no adverse changes in body weight (Supplemental Figure 8B) and no evidence of multifocal CAR-T infiltration into normal tissues or apparent tissue damage via pathological evaluation at the 2 time points evaluated (data not shown).

**Figure 5:**
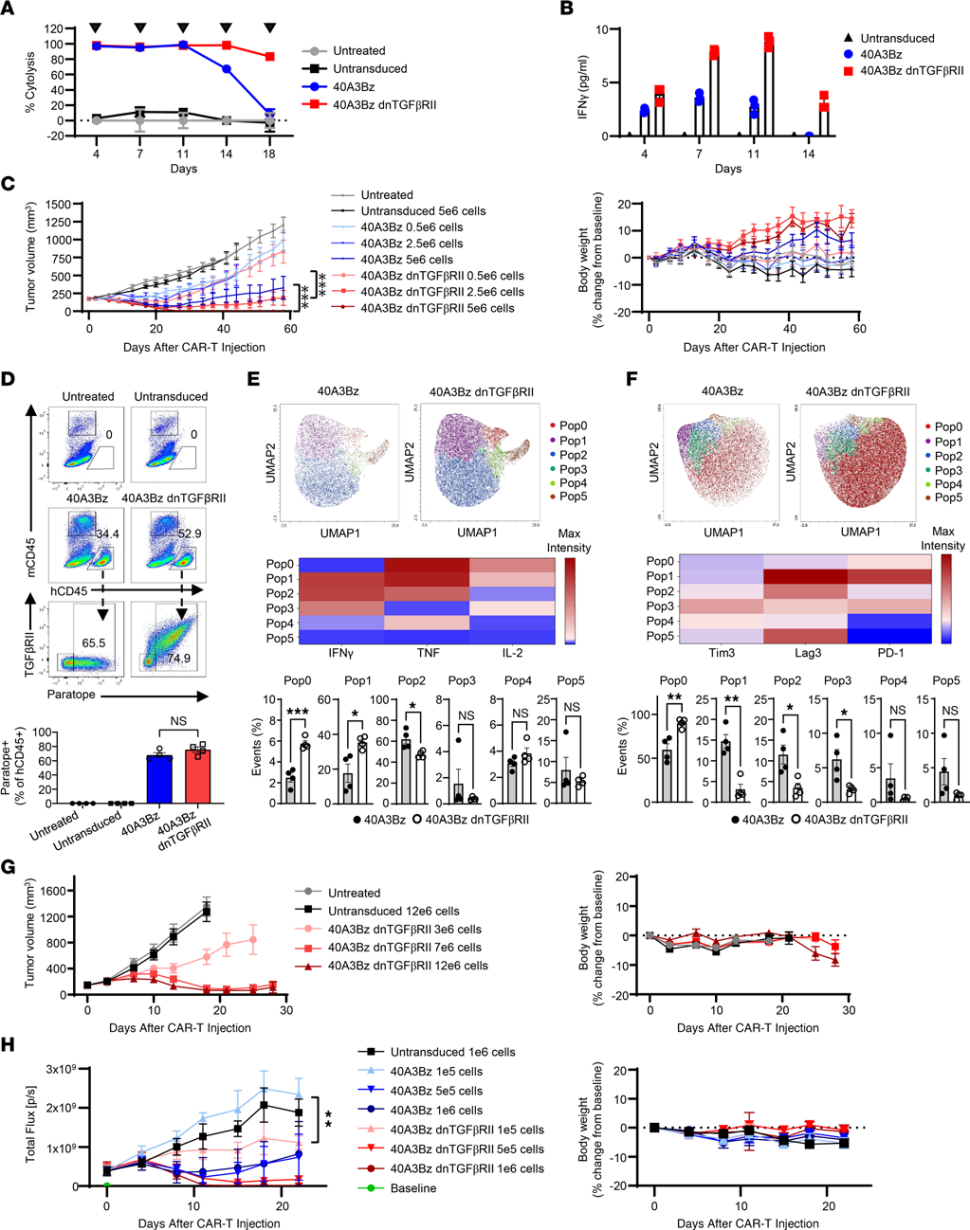
dnTGFβRII CAR-T armoring enhances activity and persistence in vitro and in vivo. (**A**) Serial killing was measured following coculture of LNCaP and CART-T cells at an E/T ratio of 0.3:1, as indicated by arrowheads. (**B**) Cytokines were profiled 24 hours after each coculture in **A**. (**C**) The 40A3Bz and 40A3Bz CAR-Ts were dosed at 3 concentrations (0.5 × 10^6^, 2.5 × 10^6^, and 5 × 10^6^ CAR-positive cells) by tail vein injection in NSG mice implanted with C4-2 cells overexpressing TGF-β (*n* = 10). Tumor volumes and body weights were measured. (**D**) For **C**, a cohort of animals from each group were sacrificed to examine CAR-T pharmacodynamics and phenotype in dissociated tumors by FACS (*n* = 4). Representative plots revealed the percentage of tumor-infiltrated human CD45^+^ (hCD45^+^) cells in mice dosed with 5 × 10^6^ cells at day 14. Bottom bar graph shows CAR-positive, tumor-infiltrated 40A3Bz cells (paratope^+^ TGFβRII^–^ hCD45^+^) and 40A3Bz dnTGFβRII CAR-Ts (paratope^+^ TGFβRII^+^ hCD45^+^). (**E**) Paratope-positive cells from **D** were analyzed for the percentage of IFN-γ^+^, TNF^+^, and IL-2^+^ cells in mice at day 14. (**F**) As in **E**, cells were analyzed for expression of Tim3^+^, Lag3^+^, and PD-1^+^ cells. UMAP plots in **E** and **F** show populations identified by a FlowSOM algorithm and further defined by heatmap. (**G**) Similarly to **C**, mice bearing 22RV1 cells overexpressing TGF-β, and 40A3Bz dnTGFβRII CAR-Ts were dosed at 3 concentrations (3 × 10^6^, 7 × 10^6^, and 12 × 10^6^ CAR-positive cells) (*n* = 10). (**H**) C4-2 luciferase-expressing cells were implanted in the intratibial space of NSG mice, and luciferase signal was monitored. Randomization occurred when the tumor flux reached 4.04 × 10^8^ photons/second (p/s), and CAR-Ts from **C** were dosed at 3 concentrations (0.1 × 10^6^, 0.5 × 10^6^, and 1 × 10^6^ CAR-positive cells). (*n* = 5). Data shown in **A**–**C** and **G** are representative of 3 independent experiments using CAR-Ts from 2 donors. **H** and **D**–**F** were performed twice with material from 2 donors. All error bars represent mean ± SEM. Statistical significance was determined using 1-sided growth rate comparison nonparametric test in **C** and **H** and unpaired 2-tailed Student’s *t* test in **E** and **F** (*P < 0.05, **P < 0.01, ***P < 0.001).

Previous reports in the context of the PSMA dnTGFβRII CAR-Ts (17) indicated that armoring may bolster CAR-T proliferation and persistence of cytotoxic activity. Thus, we performed a serial killing assay in which 40A3Bz cells and 40A3Bz dnTGFβRII CAR-Ts were repeatedly cocultured with antigen-positive LNCaP cells every 3–4 days for 5 cycles. The armored CAR-Ts maintained greater than 90% cytolysis throughout the entire assay, in contrast to the 40A3Bz cells, whose cytolytic capacity began to diminish after the third round of killing (Figure 5A). Armoring also resulted in higher cytokine production throughout the assay in comparison with 40A3Bz CAR-Ts (Figure 5B).

Because these prostate cancer cell lines produce undetectable levels of active TGF-β in in vitro culture, a TGF-β–overexpressing C4-2 line was created. This cell line was found to produce approximately 6,000 pg/mL active TGF-β in cell line–conditioned media by ELISA (data not shown) and was used in an in vivo xenograft study to evaluate the impact of dnTGFβRII armoring on CAR-T activity. This efficacy study clearly demonstrated the enhanced activity of 40A3Bz dnTGFβRII cells over the 40A3Bz CAR-Ts at each dose level in the presence of TGF-β in the TME. The improved activity of the armored CAR-Ts was also evident in the proportion of complete responders in each treatment group, with 1, 6, and 10 of 10 animals in the 0.5 × 10^6^, 2.5 × 10^6^, and 5 × 10^6^ 40A3Bz dnTGFβRII CAR-T–treated groups, respectively, exhibiting complete responses as defined by a tumor volume of 0 mm^3^ for 2 consecutive measurements. No notable changes in body weight were evident in this study (Figure 5C). At day 14 in this model, animals were sacrificed in parallel cohorts and tumors were dissociated and analyzed by FACS for CAR-T presence, phenotype, and function. In the treatment groups receiving a dose of 5 × 10^6^ cells, tumor-infiltrating human CD45^+^ cells were subsequently gated for paratope positivity to confirm CAR-T infiltration (Figure 5D). Of the paratope-positive tumor-infiltrating cells, 40A3Bz cells revealed functional activation as shown by the induction of IFN-γ^+^TNF^+^ cells (Pop2), whereas 40A3Bz dnTGFβRII CAR-Ts displayed functional activation and proliferation as shown by induction of IFN-γ^+^TNF^+^IL-2^+^ (Pop1) and TNF^+^IL-2^+^ cells (Pop0) as assessed by uniform manifold approximation and projection (UMAP) analysis (Figure 5E). Furthermore, the 40A3Bz dnTGFβRII CAR-Ts were found to have markedly reduced expression of Tim3, Lag3, and PD-1 (Pop1–3), indicating a less exhausted phenotype than 40A3Bz CAR-Ts (Figure 5F).

To understand whether 40A3Bz dnTGFβRII CAR-Ts would show antitumor activity in a lower, more heterogeneous STEAP2-expressing 22RV1–TGF-β model, xenograft tumor–bearing mice were dosed with 3 × 10^6^, 7 × 10^6^, or 12 × 10^6^ CAR-Ts per mouse. As expected, higher doses of CAR-Ts were required to see activity, and no complete responses were evident even at the highest dose of 12 × 10^6^ cells (Figure 5G). Finally, to confirm that CAR-Ts could infiltrate and enforce antitumor activity in the metastatic bone TME, a C4-2 luciferase intratibial tumor model was developed in NSG mice. Tumor measurement was monitored by luciferase detection, using the IVIS system (Xenogen). Based on prior tumor-forming dose studies, it was determined that 1 × 10^6^ C4-2 luciferase cells would be implanted intratibially and lead to tumor formation to allow randomization at 17 days. Upon randomization, 1 × 10^5^, 5 × 10^5^, and 1 × 10^6^ 40A3Bz cells and 40A3Bz dnTGFβ CAR-Ts and 1 × 10^6^ untransduced control T cells were administered to mice by tail vein. A higher degree of tumor regression and complete responses was evident in the 40A3Bz dnTGFβRII–treated groups than in the 40A3Bz-treated groups, thereby confirming efficient antitumor activity of the armored product in the setting of the bone TME (Figure 5H).

**Figure 6:**
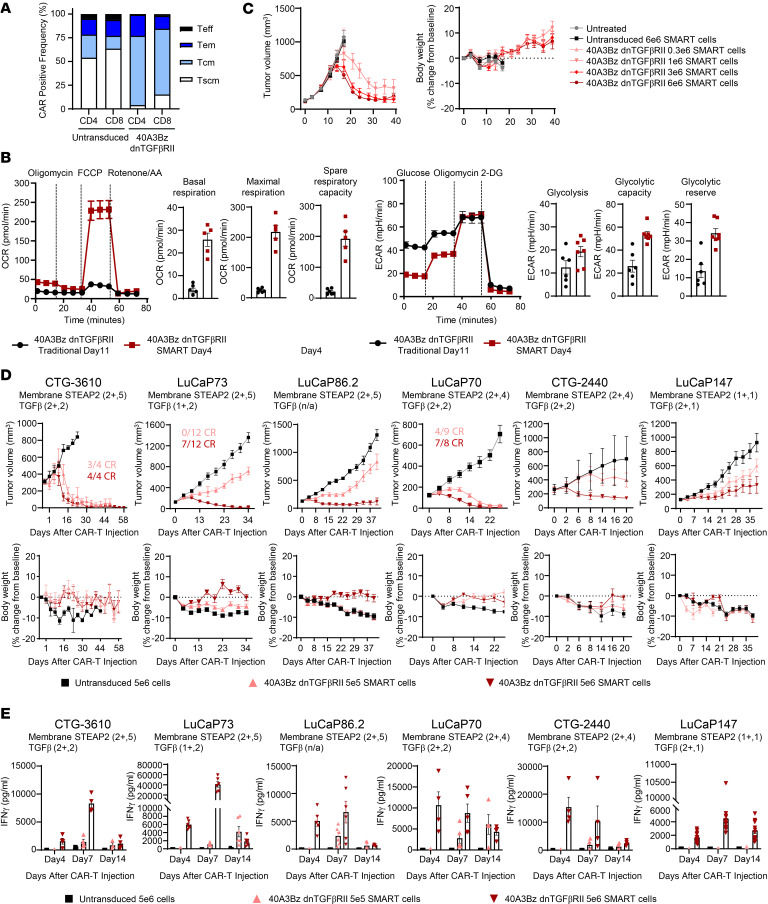
Effect of enhanced CAR-T manufacturing on antitumor activity. (**A**) 40A3Bz dnTGFβRII CAR-Ts were manufactured according to the SMART process, and CAR positivity, activation, and phenotype of the cells were evaluated at expansion day 4 and compared with those of untransduced T cells from the same donor. (**B**) Bioenergetic profile of SMART (day 4) versus traditional (day 11) manufactured 40A3Bz dnTGFβRII CAR-Ts as determined by Seahorse analysis. OCR, oxygen consumption rate; ECAR, extracellular acidification rate. (**C**) 40A3Bz dnTGFβRII SMART CAR-Ts were dosed at 4 concentrations (0.3 × 10^6^, 1 × 10^6^, 3 × 10^6^, and 6 × 10^6^ CAR-positive cells) by tail vein injection in NSG MHC class I/II knockout mice implanted with 22Rv1 cells overexpressing TGF-β (*n* = 10). Tumor volumes and body weights were measured to 50 days after tumor implantation. (**D**) PDX fragments from frozen stocks of various prostate cancer PDX models were implanted into NSG MHC I/II knockout mice and randomized when tumor volumes for each model ranged from 125 to 250 mm^3^. Mice were dosed as described in **C** with 0.5 × 10^6^ or 5 × 10^6^ 40A3Bz dnTGFβRII SMART CAR-Ts and compared with 5 × 10^6^ untransduced SMART controls (*n* ranged from 4 to 12 depending on the model). The IHC data inset on each model represents the cell surface STEAP2 expression scoring. (**E**) Serum levels of IFN-γ across all PDX models described in **D**, determined by MSD (*n* = 4 or greater). Experiments are representative of 2 different donor CAR-Ts. Data in **A**–**C** are representative of multiple independent experiments prepared from 3 healthy donors, while **D** and **E** were performed once with material from 1 donor. All data represent mean ± SEM.

Next, we explored the possibility that a shorter CAR-T manufacturing period would yield a less differentiated CAR-T product, which may improve the ability of 40A3Bz dnTGFβRII CAR-Ts to engraft and persist after infusion (18) (YTB323 versus tisagenlecleucel). We named this the shortly manipulated actively replicating T cell (SMART) platform. In this process, T cells were expanded for 4 days in the presence of 40 IU/mL IL-2 and 0.24 U/mL IL-21, and the CAR-positive cells contained a large fraction of central memory T cells and memory stem T cells as previously defined by CD62L and CD45RO FACS parameters in Figure 4D (Figure 6A). To determine the impact of the manufacturing process on the CAR-T cell metabolic profile, SMART and traditional 40A3Bz dnTGFβRII CAR-Ts were compared by Seahorse Cell Mito Stress and Glycolytic Stress tests. In this assay spare respiratory capacity is defined as a quantitative difference between the maximal and basal oxygen consumption rates and represents the extra mitochondrial capacity available in the cell to produce energy under stress. SMART CAR-Ts exhibited higher basal and maximal respiration, as well as increased spare respiratory capacity, compared with the traditional CAR-Ts. Additionally, the extracellular acidification rate was measured, which quantifies glycolytic flux in response to glucose and oligomycin (an ATP synthase inhibitor that drives cells to maximal glycolytic activity by shutting down oxidative phosphorylation). Consistent with the previous assay, SMART CAR-Ts exhibited heightened glycolysis, glycolytic capacity, and glycolytic reserve compared with traditional CAR-Ts (Figure 6B). To understand the impact of this favorable metabolic profile on antitumor activity and persistence in vivo, 40A3Bz dnTGFβRII SMART CAR-Ts were administered at doses of 0.3 × 10^6^, 1 × 10^6^, 3 × 10^6^, and 6 × 10^6^ in an efficacy study in the 22RV1–TGF-β model, similar to the study described in Figure 5G, but to postpone the onset of graft-versus-host disease, NSG MHC class I/II–deficient mice were used. In line with our hypotheses, antitumor activity was achieved at lower doses with the SMART cell product without any overt toxicity (Figure 6C). Finally, as there is a lack of data in the literature supporting the efficacy of prostate-directed CAR-T monotherapy in patient-derived xenograft (PDX) models, we aimed to interrogate the activity of the SMART-manufactured 40A3Bz dnTGFβRII CAR-Ts in heterogeneous prostate PDX tumor models that are more representative of the patient setting. We tested CAR-T efficacy in 6 prostate PDX models of varied 1 and 2+ cell surface STEAP2 expression levels by IHC, and in every model, dose-dependent tumor growth inhibition and/or regression (Figure 6D), with accompanying IFN-γ release (Figure 6E), was achieved without signs of toxicity as assessed by body weight. To interrogate CAR-T persistence in a model that demonstrated a dose-dependent response, CAR-Ts were detected by FACS in the whole blood and spleen of LuCaP 86.2 tumor–bearing animals at day 42 (Supplemental Figure 9). As expected, CAR-Ts were detected in a dose-dependent manner even after tumor clearance.

## Discussion

In prostate cancer there is a paucity of optimal tumor antigens that display high homogeneous expression across the tumor and limited expression in normal tissues. Many publications have underscored the attractiveness of STEAP2 for prostate cancer targeting, not only because of its favorable expression profile, but also because it is reported to be a driver of prostate cancer proliferation, indicating that it may not be easily dispensable as a means of resistance (6, 19, 20). To our knowledge, the work presented here highlights the first preclinical therapeutic that targets STEAP2. Taken together, the results validate STEAP2 as an ideal prostate tumor antigen displaying high, largely homogeneous expression throughout all stages of disease progression, as well as cell surface distal, normal tissue expression confined to the prostate. This expression profile was thoroughly confirmed through orthogonal methods at the RNA level via ISH, through IHC using an antibody with a distinct epitope to the therapeutic, and through antibody-independent means using targeted proteomic evaluation of critical human healthy tissues. In addition, we designed a human/murine cross-reactive, STEAP2-specific, potent, and tolerable CAR-T that demonstrated antitumor activity in STEAP2-positive cell line xenografts. This in vivo activity was evident even in the challenging contexts of the bone TME and heterogeneous low- to moderate-STEAP2-expressing PDXs. Together, with the rational and clinically tested strategies of dnTGFβRII armoring (15) and shortened manufacturing (21, 22), STEAP2 targeting can be expected to enable enhanced CAR-T persistence and durable responses.

Although several other prostate antigens, such as PSMA and PSCA, are being tested clinically with T cell–engaging bispecific antibodies, CAR-Ts, and antibody-drug conjugates, the field is currently learning about the suitability of these targets and the efficacy of these modalities in the prostate cancer setting. However, it could be envisioned that STEAP2 CAR-Ts may provide a superior approach for multiple reasons. First, STEAP2 displays an ideal expression profile for targeting as compared with many other prostate tumor antigens due to limited distal normal tissue expression, as evaluated by IHC (Table 1), the Human Protein Atlas, and proteomic data sets. Thus, the restricted expression profile of STEAP2 may minimize the risk of off-tumor, on-target sink tissues and potential toxicities, compared with the gastrointestinal tract, brain, liver, and kidney expression seen with PSMA (10, 23–26). Second, advanced prostate cancer has been considered an immunologically “cold” tumor type owing to the immunosuppressive forces in the bone microenvironment (13, 27). However, it may be that CAR-Ts can introduce a T cell–mediated immunological response in tumors with considerable immunosuppressive barriers. This has been demonstrated nonclinically for PSMA- and PSCA-targeted CAR-T therapies, and this hypothesis has been under recent clinical evaluation in the TM-PSMA-01 and the MB 105 (PSCA-targeting CAR-T technology) studies (15, 17, 28, 29).

Beyond target expression, additional factors that could endow AZD0754 with an advantage over previous prostate-directed CAR-Ts are contributed by the dnTGFβRII armoring that augments persistence in the immunosuppressive TME, as well as a shortened manufacturing process that enriches for phenotypically “younger” cells with enhanced fitness. CAR-Ts generated from our SMART manufacturing process are inherently less prone to elicit CRS because of reduced levels of early T cell activation as well as reductions in monocyte activation and cytokine release, thereby contributing to the hypothesized improved safety profile of this approach. Taken together, our results suggest that a STEAP2-targeted CAR-T product may have the potential to provide a wider therapeutic index than previous prostate-directed immune-oncology approaches for patients in need of new therapies. Finally, the exquisite sensitivity of the intratibial in vivo tumors (Figure 5H) as compared with the subcutaneous model (Figure 5C) with the same batch of CAR-Ts allows us to question whether tumors in the bone microenvironment could be more responsive to CAR-T therapy because of improved access to tumor from the vasculature, lower levels of stromal barriers, and/or enhanced cytokine signaling. This has been observed in a rhabdomyosarcoma patient with bone marrow disease and relapse who had a favorable response to HER2 autologous CAR-T therapy (30). Further work is necessary to test this idea, which could provide a compelling reason to consider the use of CAR-T therapies in metastatic prostate cancer.

Clinical evidence from studies of CAR-T therapy in hematopoietic malignancies has suggested that improved responses to therapy will correlate to target homogeneity and target biology. Encouragingly, our STEAP2 IHC data in tumor samples indicated high homogeneity of the target, as 93% of CRPC, 89% of lymph node metastatic, and 85% of bone metastatic patient samples showed ≥75% of the tumor-expressing membrane STEAP2 (Figure 2B). While it is difficult to compare expression levels of various targets because of differing tumor samples, IHC protocols, and reagents, cell surface STEAP2 expression seems to be equivalent to or higher than the expression reported for other clinically tested prostate targets, including PSCA, PSMA, and STEAP1 (31–32). Although the biology of STEAP2 regulation and function remains largely unstudied, there is evidence in the literature that STEAP2 increases prostate cancer cell growth and invasion (34, 35). Further, STEAP2 expression correlates with Gleason score and can even be used as a diagnostic marker to predict patient prognosis (8). In support of the idea that STEAP2 is a critical driver of prostate cancer, our preclinical studies did not reveal evidence of target loss as a means of resistance to therapy. This is challenging to evaluate in some in vivo studies owing to the lack of viable tumor tissue at the end of study, but in the CTG-2440 and LuCaP 73 models dosed at the 5 × 10^5^ cell sub-efficacious levels (Figure 6D), we observed consistent STEAP2 IHC scoring before and after CAR-T dosing. This result contrasts with the findings from recent reports on a STEAP1 CAR-T therapy (31, 36), which perhaps underscores the differences in targeting these 2 proteins, which could be attributable to greater STEAP2 target homogeneity or to the functional N-terminus of STEAP2 that is critical for prostate cell survival but is absent in STEAP1. In line with the latter observation, our generation of a viable clonal LNCaP STEAP2 CRISPR cell line was challenging, requiring 3 rounds of CRISPR knockout. Despite undetectable levels of STEAP2 protein, the LNCaP STEAP2 CRISPR cell line maintained 26% wild-type allele and manifested substantial STEAP1 overexpression that was likely developed by the cell to compensate for STEAP2 reductions (data not shown).

Limitations of this study stem in part from the challenging protein structure of STEAP2. As such, our tumor and distal normal tissue expression data are built upon ISH, IHC, and targeted proteomic data sets. ISH and targeted proteomic analyses identified low levels of STEAP2 transcript and protein in non-prostate normal tissues. Because of the inherent risk in targeting a novel antigen with an exquisitely potent CAR-T therapy, we are currently elaborating on the human normal tissue expression pattern via additional cell surface proteomic experiments, which may help further define very low levels of target expression that could pose an on-target, off-tumor toxicity risk in a clinical setting. Furthermore, although 40A3 is murine cross-reactive, we are still developing our understanding of cell surface murine STEAP2 expression in tissues to identify potential toxicities, as well as building the models to evaluate the impact of human cytokines on murine tissues to evaluate CRS. An additional limitation of the data herein is the difficulty in accurately quantifying and modeling the level of active TGF-β in the bone tumor setting in the patient. While the level of TGF-β in the C4-2 TGF-β cell line supernatant (6,000 pg/mL) is in line with reports in murine bone tumor models (~2,000 pg/mL), it could underestimate the levels present in the TME in prostate cancer patients, which have been reported to be as high as 25,000 pg/mL (13). To address this potential difference, in Figure 4H the cytotoxic activity of AZD0754 in the presence of 30,000 pg/mL recombinant TGF-β was confirmed. Finally, our murine models do not take into account the host immune system and cannot identify potentiation of CAR-T–mediated cytotoxicity in the inflamed TME. To address this limitation, we are performing studies with murine T cells containing the 40A3 CAR construct with murine costimulatory domains and murine dnTGFβRII. These studies in the immunocompetent setting containing murine STEAP2-overexpressing tumors will allow us to evaluate the impact of lymphodepletion and host immune cell activity on the safety and efficacy of STEAP2 targeting. Using mouse surrogate immune-oncology agents, we plan to perform combination studies to identify clinically relevant strategies to prime the TME as well as to improve CAR-T function and persistence.

Collectively, the results of these studies demonstrate that the STEAP2 protein structure, expression profile, and biology are therapeutically tractable and support further evaluation of this target in prostate cancer. The promising results from our preclinical studies with 40A3Bz dnTGFβRII CAR-T therapy, AZD0754, warrant its continued testing and development in human clinical trials evaluating safety and efficacy of this CAR-T therapy in patients with metastatic prostate cancer.

## Methods

### Cell culture and cell line generation

Cells were obtained from the American Type Culture Collection (ATCC) and grown according to the vendor’s recommendations, with the exception of C4-2, which was obtained from G.N. Thalmann at the University of Bern, Bern, Switzerland. Cells were grown at 37°C, 5% CO_2_ in a humidified incubator. Cell line authentication was conducted by short tandem repeat–based DNA fingerprinting and multiplex PCR, and mycoplasma negativity was verified. To ensure similarity of cell passage throughout experiments, cells were cultured for fewer than 6 passages and banked. Human primary cells (ScienCell Research Laboratories) were cultured according to the vendor’s recommendations, and not passaged. The STEAP3-2 human, murine, and loop-swapped chimeras were recombinantly generated by replacement of the extracellular loops of STEAP3 with the corresponding human, murine, or individual loop STEAP2 sequences, whereas the remainder of the DNA was STEAP3 native sequence. The STEAP3-2 gene C-terminally fused with a Snorkel-FLAG tag was inserted into a pCDH-CMV vector using restriction enzymes XbaI and NotI. Stable chimera-expressing cells were created using lentivirus expression vectors produced through the pPACKH1-XL packaging mix (System Biosciences), transduction, and puromycin selection. Cell surface expression was assessed and confirmed by staining with an anti-FLAG M2 antibody (Thermo Fisher Scientific). The following stable cell lines were created by the same lentivirus method: Ad293 STEAP1, Ad293 STEAP3, Ad293 STEAP4, 22RV1 TGF-β, C4-2 TGF-β, and C4-2 firefly luciferase. The LNCaP-STEAP2–clustered regularly interspaced short palindromic repeats (CRISPR) stable knockout line was created using a combination of Alt-R gRNA09 (Integrated DNA Technologies) and 3 pooled STEAP2 guide RNAs (Gene Knockout Kit, version 2; Synthego) and was generated according to the manufacturer’s recommendations. The knockout line was validated by Western blot, flow cytometry, and amplicon sequencing.

### In situ hybridization and immunohistochemistry

A rabbit polyclonal antibody campaign was run against a C-terminal peptide of human STEAP2 to generate the ME668 polyclonal anti-STEAP2 antibody used in immunohistochemistry (IHC). STEAP2 IHC staining was performed on formalin-fixed, paraffin-embedded (FFPE) samples on a Dako instrument (Agilent). Briefly, samples were deparaffinized with 95% ethanol, and endogenous peroxidase block was applied before unmasking in the pressure cooker with Dako Target retrieval solution. Samples were then subjected to a fish gelatin blocking solution, primary antibody (0.3 μg/mL), Dako anti-rabbit–horseradish peroxidase secondary antibody, and ImmPACT 3,3′-diaminobenzidine substrate (Vector Laboratories), with 3 washes between steps, and finally hematoxylin and bluing reagent. Slides were imaged on an Aperio slide scanner (Leica Biosystems). Grading of staining was performed at AstraZeneca according to a scoring system that incorporated intensity and heterogeneity, using the following IHC intensity score: 0, no staining; 1, low; 2, medium; 3, high. The proportion of tumor with staining by IHC was scored as follows: 0, no staining; 1, 0%–20%; 2, 20%–40%; 3, 40%–60%; 4, 60%–80%; 5, >80%. Tumor microarrays (TMAs) were graded by quantitative continuous scoring (QCS) and reviewed by a licensed pathologist (37). All normal tissue arrays and TMAs were purchased from US Biomax. In situ hybridization (ISH) was performed with a human STEAP2 probe on FFPE TMAs at Advanced Cell Diagnostics and was scored according to the following criteria: 0, no staining or <1 dot per 10 cells; 1, 1–3 dots per cell; 2, 4–9 dots per cell with no or very few dot clusters; 3, 10–15 dots per cell with <10% dot clusters; 4, >15 dots per cell with >10% dot clusters.

### Targeted mass spectrometry

#### Laser microdissection.

Laser microdissection was carried out as previously described (38) on pathologist-annotated H&E-stained slides using an MMI CellCut (Molecular Machines and Industries) equipped with an ultraviolet laser.

#### Sample preparation.

Microdissected tissues were collected in low-bind tubes and dissolved in 2% sodium dodecyl sulfate, 40 mM chloroacetamide, 10 mM tris(2-carboxyethyl)phosphine, and 100 mM tris(hydroxymethyl)aminomethane (Tris), pH 8, before incubation at 95°C with shaking at 1,000 rpm for 90 minutes in an Eppendorf ThermoMixer C (Sigma-Aldrich). Samples were transferred to a 96-well plate (Abgene SuperPlate, AB2800, Sigma-Aldrich) and transferred to a Bravo automation system for SP3-bead–based sample preparation (39). Briefly, Sera-Mag carboxylate beads (GE24152105050250 and GE44152105050250, Sigma-Aldrich) and acetonitrile were added to the samples. After agitation, the samples were transferred to a magnetic plate (Magnum FLX, A000400, Alpaqua Engineering) to remove supernatant. The beads were washed twice with 80% ethanol, once with acetonitrile, and were resuspended in 100 mM Tris. Trypsin (V5111, Promega) was added, and the samples were incubated at 37°C at 1,000 rpm for 18 hours in the ThermoMixer. Samples were subsequently acidified with trifluoroacetic acid at a final concentration of 0.5%. The supernatant was desalted using a reversed-phase sorbent (Oasis HLB 96-well μElution Plate, 186001828BA, Waters) with elution in 70 μL of 50% acetonitrile with 0.1% formic acid. Samples were dried by vacuum centrifugation and resuspended in aqueous 0.1% formic acid, shaking at 50°C at 1,000 rpm for 10 minutes. Peptide concentration was determined using a bicinchoninic acid assay (Micro BCA Protein Assay Kit, 23235, Thermo Fisher Scientific) with 10% radioimmunoprecipitation buffer as diluent (89900, Pierce Chemical).

#### FAIMS-PRM.

Targeted mass spectrometry was carried out as previously described (40). Samples (0.5 μg, spiked with 5 fmol of isotope-labeled synthetic peptides) were loaded onto EvoTip trapping columns (EV2015, EvoSep) before separation with the EvoSep One nanoLC system coupled to an Orbitrap Exploris 480 mass spectrometer with a FAIMS Pro interface (Thermo Fisher Scientific). Peptides were eluted over a 44-minute gradient, from 7% to 30% acetonitrile (on-column) at a flow rate of 500 nL/min. The mobile phase comprised buffer A, 0.1% aqueous formic acid (9834-02, JT Baker), and buffer B, 0.1% formic acid in acetonitrile (9832-02, JT Baker). The analytical column was 15 cm × 100 μm with 3 μm particles (15-100-3-UHPnC, PepSep). A stainless steel emitter was used (30 × 150 μm; PSSELJ, PepSep) with an applied voltage of 1.9 kV. The high-field asymmetric waveform ion mobility spectrometry (FAIMS)–parallel reaction monitoring (PRM) experiment used high-energy collisional dissociation fragmentation with an isolation window of 0.7 mass-to-charge ratio (*m/z*), a standard automatic gain control, and a maximum injection time of 118 milliseconds. Tandem mass spectrometry scans were acquired in profile mode, using 60K resolution at 200 *m/z*. FAIMS was operated at the standard resolution, with no additional FAIMS gas.

#### Data analysis.

PRM data were analyzed with Skyline (version 22.2.0, University of Washington, Seattle, Washington, USA) using high-selectivity extraction. Fragment ions with interference were identified by manual analysis, comparing coelution and fragment ion ratios between endogenous and reference peptides. Any fragment ions showing interference were flagged and omitted from use in quantitation. Quantitation (amol/μg) was obtained from a single fragment ion, with the amount calculated from the fragment “Area Ratio” exported from Skyline, multiplied by the internal standard amount (5 fmol) and divided by the loaded amount (0.5 μg). Lower and upper limits of quantitation (LLOQ and ULOQ) were determined from a 10-point reverse curve, with heavy synthetic peptide from 2.5 amol to 50 fmol spiked into 0.5 μg human spleen matrix, as previously described (40). Mean fragment ion accuracy extrapolated from linear regression was required to be within 80%–120% of the expected value, and the coefficient of variation was required to be less than 20%, for all points from the LLOQ to the ULOQ.

### Antibody generation

Antibodies 40A3 and 30D12 were generated in Del-1 or STEAP2-knockout mice (homozygous Steap2 K/O, Taconic Bioscience), respectively. Mice were immunized with Ad293 STEAP3-2 cells, and spleens and lymph nodes were harvested before B cell fusion with myeloma. Hybridoma supernatants were deselected using LNCaP STEAP2 CRISPR cells. Specific and murine cross-reactive clones were selected based on FACS binding to Ad293 STEAP3-2 human and murine cells and LNCaP cells and concurrent lack of binding to LNCaP STEAP2 CRISPR, Ad293, Ad293 STEAP1, Ad293 STEAP3, and Ad293 STEAP4 cells. Following cloning and sequencing, recombinant immunoglobulin G (IgG) molecules were generated. The 40A3 IgG was converted to a single-chain variable fragment (scFv) for incorporation into the CAR-T cassette. For 40A3 anti-paratope generation, 40A3 IgG and 40A3 scFv–crystallizable fragment (Fc) were injected into BL6 mice. Hybridoma supernatants were screened by FACS to identify binders to 40A3Bz cells and 40A3Bz dnTGFβRII CAR-Ts and lack of binding to untransduced cells or an unrelated CAR-T product. m11G13 was selected as a 40A3 scFv CAR-T–specific anti-paratopic antibody that competes with 40A3 IgG for STEAP2 binding.

### Western blotting

Cells were washed once with PBS and lysed by addition of Laemmli reducing buffer (6× diluted to 2× with water; Boston BioProducts). Cell lysates were collected, boiled, and then loaded onto NuPAGE 4%–12% Bis-Tris gels (Thermo Fisher Scientific). Proteins were then transferred to polyvinylidene fluoride membranes (Thermo Fisher Scientific), using an iBlot dry blotting system (Thermo Fisher Scientific). Membranes were blocked with 5% nonfat dry milk and 0.05% Tween-20 (Bio-Rad Laboratories) in Tris-buffered saline, pH 7.4, and incubated overnight at 4°C with antibodies (STEAP2 rabbit polyclonal custom-made with Covance, ME668, 1:2,000, and EGFR, 4267S, 1:1,000; Cell Signaling Technology). Membranes were washed in 0.05% Tween-20 in Tris-buffered saline and then incubated with horseradish peroxidase–conjugated streptavidin secondary antibodies (Jackson ImmunoResearch). After washing, protein bands were detected using SuperSignal West Femto Chemiluminescent substrate and SuperSignal West Pico Chemiluminescent substrate (Thermo Fisher Scientific). An ImageQuant LAS4000 instrument (GE Healthcare) was used to capture and analyze images.

### Flow cytometry

Flow cytometry (FACS) was performed by harvesting of 80% confluent cell cultures with Cell Dissociation Buffer (Thermo Fisher Scientific) and staining at 4°C with FLAG M2 antibody (Sigma-Aldrich) for 1 hour, and, if not directly conjugated to a fluorophore, a secondary anti-human–Alexa Fluor 647 antibody was used (Thermo Fisher Scientific) for 30 minutes. Cells were stained with DAPI and analyzed on a Fortessa cell analyzer (BD Biosciences). Mean fluorescence intensity values from a nonspecific control IgG1 were subtracted as background.

STEAP2 receptor density was determined by FACS with the anti-STEAP2 clone 30D12 conjugated to Alexa Fluor 647 antibody, using an Alexa Fluor 647 antibody labeling kit (Thermo Fisher Scientific) and the Quantum Simply Cellular anti-human kit (Bangs Laboratories) according to the manufacturers’ instructions. To assign antibody-binding capacity, a standard curve was generated using a bead-dilution series.

### CAR-T generation and phenotyping

Titered lentivirus was produced by Lentigen Technology, using proprietary vectors and methods according to sequences provided. The CAR genes were constructed by fusing of the following in series: human CSFR2 signal peptide, scFv, and human IgG4 hinge with S228P mutation, human CD28 transmembrane domain, human 4-1BB cytoplasmic domain, and human CD3ζ cytoplasmic domain. To generate research-grade CAR-Ts, purified T cells were seeded in AIMV medium (Gibco) containing 5% human serum (Valley Biomedical) and IL-2 (300 IU/mL; PeproTech). T cells were activated with CD3/CD28 Dynabeads (Invitrogen) according to the manufacturer’s protocol. The following day, lentivirus was added and plates were centrifuged at 2,000*g* and 37°C for 2 hours and then placed in a 5% CO_2_ tissue culture incubator. Cells were maintained at a cell density of 0.5 × 10^6^ to 1 × 10^6^ cells/mL. Transduction efficiency was determined by flow cytometry, using anti-40A3 paratope antibody (as described above) and the anti-TGFβRII antibody (W17055E, BioLegend). For the shortly manipulated actively replicating T cells (SMART) manufactured CAR-T generation, T cell activation and transduction were accomplished as before, but expansion was accomplished in shaker flasks in incubators at 37°C and 5% CO_2_ and 51 rpm shaking and harvested on day 4.

To phenotype the CAR-Ts by FACS, various antibodies were used to identify stemness, activation, and exhaustion markers. Anti–human CD4 (RPA-T4, catalog 742000), CD8 (RPA-T8, catalog 566121), CD62L (SK11, catalog 565219), CD45RO (UCHL1, catalog 562791), CD69 (FN50, catalog 562617), LAG3 (T47-530, catalog 565720), and PD-1 (EH12, catalog 564104) were purchased from BD Biosciences. Anti–human CD45 (HI30, catalog 304034) and TIM3 (F38-2E2, catalog 345022) were obtained from BioLegend. Dead cells were excluded by staining with a Live/Dead Fluorescent Blue Dead Cell Stain Kit (Invitrogen) for 15 minutes on ice in PBS. Surface markers were detected by incubation of cells with relevant monoclonal antibodies for 30 minutes on ice. Samples were acquired on an LSR Fortessa or LSR II instrument (BD Biosciences) and analyzed with FlowJo software, version 10.1 (Tree Star).

### CAR-T in vivo intratumor characterization

Tumors were dissociated in buffer containing collagenase type IV (Sigma-Aldrich), DNase I (Sigma-Aldrich), and hyaluronidase (Sigma-Aldrich). Single-cell suspensions were generated using GentleMACS Octo dissociator (Miltenyi Biotec). Dead cells were excluded by staining with a Live/Dead Fluorescent Blue Dead Cell Stain Kit (Invitrogen) for 15 minutes on ice in PBS. The following antibodies were used for surface staining: anti–human CD45 (HI30, catalog 304034, BioLegend), anti–mouse CD45 (30-F11, catalog 566439, BD Biosciences), paratope (in house), anti–human TGFβRII (W17055E, catalog 399704, BioLegend), anti–human Lag3 (T47-530, catalog 565720, BD Biosciences), anti–human Tim3 (F38-2E2, catalog 345022, BioLegend), and anti–human PD-1 (EH12, catalog 564104, BD Biosciences). For intracellular cytokine staining, the cells were stimulated for 4 hours in phorbol 12-myristate 13-acetate (20 ng/mL) and ionomycin (1 μg/mL) in the presence of protein transport inhibitor GolgiPlug (BD Biosciences) containing brefeldin A (1:1,000 dilution). Thereafter, cells were surface-stained, washed, and fixed using Cytofix/Cytoperm buffer (BD Biosciences). Intracellular staining was performed with the following antibodies: anti–human IFN-γ (B27, catalog 506538, BioLegend), anti–human TNF (MAb11, catalog 502948, BioLegend), and anti–human IL-2 (MQ1-17H12, catalog 500326, BioLegend). Flow cytometry analysis was performed using FlowJo 10.7.1 plug-ins. Dimensionality reduction and marker visualization/clustering were performed using uniform manifold approximation and projection (UMAP) and FlowSOM algorithms. Cluster Explorer (https://www.flowjo.com/exchange/#/plugin/profile?id=30) was used to generate plots based on FlowSOM clustering and illustrates those populations overlaid on UMAP visualization. Samples were acquired on a FACSymphony A5 instrument (BD Biosciences) and analyzed using FlowJo software, version 10.7.1.

### CAR-T functional assay

Cytotoxicity was analyzed across a panel of cell lines, wherein target cells were seeded into 96-well E-plates (Acea Biosciences) at 1 × 10^4^ to 4 × 10^4^ cells per well (depending on cell line growth kinetics) in ATCC-recommended cell line medium, and cell index was monitored with the xCELLigence impedance-based real-time cell analysis system (Acea Biosciences). The next day, CAR-Ts were resuspended in cell medium and added to the coculture. Points were performed in triplicate.

To analyze in vitro cytokine secretion, 25 μL of supernatant was removed from the cocultures at 24 hours and analyzed by Meso Scale Discovery (MSD) assay (Meso Scale Diagnostics) according to the manufacturer’s instructions.

### CAR-T serial cytotoxicity

A continuous in vitro coculture assay was used to evaluate the target-killing ability and persistence capacity of CAR-Ts after multiple rounds of antigen challenge. On day 0, CAR-Ts and LNCaP cells were plated at an effector-to-target (E/T) ratio of 0.3:1 and incubated for 3 days. CAR-Ts were then rechallenged with fresh LNCaP cells every 3–4 days. After each round of rechallenge, CAR-Ts were counted to determine expansion, spun down at 500*g* for 5 minutes, and then resuspended in fresh medium. Dead cells were removed by magnetic separation (Miltenyi Biotec) before each rechallenge. Culture supernatants were collected at 24 and 72 hours after each challenge. IFN-γ levels were measured by ELISA (R&D Systems). At the end of each antigen challenge, flow cytometry was performed to determine CAR-T activation, differentiation, and exhaustion as described above. LNCaP cell killing was determined using CellTiter-Glo reagent (Promega).

### Bioenergetic analyses

After thawing, CAR-Ts were resuspended and incubated in X-VIVO 15 medium (Lonza Bioscience) for an hour. Cells were stained with 1 μg/mL paratope–Alexa Fluor 647 at room temperature for 10 minutes, and the STEAP2 CAR-positive T cells were isolated using Anti-Cy5/Anti–Alexa Fluor 647 Microbeads (Miltenyi Biotec) following the manufacturer’s instructions. For Seahorse analyses, cells were plated onto 96-well Seahorse XFe96 PDL cell culture microplates (0.2 × 10^6^ cells per well) and coated with poly-d-lysine to facilitate T cell attachment. The glycolysis stress test (103017-100, Agilent Technologies) was performed by measurement of extracellular acidification rate (milli-pH/min) at steady state and after sequential injection of d-glucose (10 mM), oligomycin (1 μM), and 2-deoxy-d-glucose (50 mM) (all Agilent Technologies). The mitochondrial stress test (103010-100, Agilent Technologies) was performed by measurement of oxygen consumption rate (pmol/min) at steady state and after sequential injection of oligomycin (1.5 μM), FCCP (2 μM), rotenone, and antimycin A (0.5 μM) (all Agilent Technologies). Experiments were run on a Seahorse XFe96 instrument with the following assay conditions: 3 cycles of 3-minute mixture and 3-minute measurement.

### In vivo efficacy models

Cell line–derived xenograft models were developed by subcutaneous injection of a 1:1 ratio of tumor cell line to Matrigel (Corning Life Sciences) or Cultrex (R&D Systems) mixture into the flanks of 5- to 7-week-old male NSG (NOD.Cg-Prkdc^scid^ Il2rg^tm1Wjl^/SzJ) or male NSG MHC class I/II double-knockout (NOD.Cg-Prkdc^scid^ H2-K1^tm1Bpe^ H2-Ab1^em1Mvw^ H2-D1^tm1Bpe^ Il2rg^tm1Wjl^/SzJ) mice (The Jackson Laboratory) as indicated. Resulting tumors were measured twice per week, and tumor volume was calculated by the formula: tumor volume (mm^3^) = (length × width^2^)/2. Mice were randomized when tumor volumes had reached approximately 175 mm^3^, and CAR-Ts were administered once intravenously at the specified dose. Tumor growth was monitored at least twice weekly. Where statistics are provided, growth rate comparisons were run as previously described (41). The orthotopic bone metastatic tumor model was implanted by dilution of 1 × 10^6^ C4-2 luciferase cells in 10 μL of PBS and injection of the cells into the intratibial space in the rear hind limb, and noninvasive optical imaging with an IVIS intravital imaging system (Xenogen) was performed according to the manufacturer’s instructions. Flux signals were obtained biweekly and analyzed with Living Image software (Xenogen).

For serum cytokine analysis, peripheral blood was harvested at the indicated times, and serum was separated with Microtainer Serum Separator tubes (BD Biosciences). Cytokine levels were determined by MSD assay according to the manufacturer’s instructions.

Prostate cancer patient-derived xenograft (PDX) models were chosen based on STEAP2 gene expression and IHC analysis. In vivo studies of CAR-T activity were carried out in PDX models at Champions Oncology, and LuCaP models (73, 86.2, 70, and 147) were tested at AstraZeneca according to standard procedures. PDX models were developed by subcutaneous implants of PDX fragments into the flanks of 5- to 7-week-old male NSG MHC class I/II double-knockout (NOD.Cg-Prkdc^scid^ H2-K1^tm1Bpe^ H2-Ab1^em1Mvw^ H2-D1^tm1Bpe^ Il2rg^tm1Wjl^/SzJ) mice (The Jackson Laboratory). To match the clinical scenario, all animals in the in vivo studies were given CAR-Ts that had been previously frozen.

### Statistics

Significant differences between experimental groups were calculated using the 1-sided growth rate comparison nonparametric test or using unpaired 2-tailed Student’s *t* test. Values of *P* less than 0.05 were regarded as statistically significant. Data analysis was performed using R or GraphPad Prism (version 9.5.1) software.

### Study approval

All procedures involving animals were conducted in facilities accredited by the Association for Assessment and Accreditation of Laboratory Animal Care International and were approved by the AstraZeneca Institutional Animal Care and Use Committee, in accordance with the Institute for Laboratory Animal Research’s *Guide for the Care and Use of Laboratory Animals* (8th edition, National Academies Press, 2011) and AstraZeneca’s Bioethics Standard.

### Data availability

Data included in this article are provided in the Supporting Data Values file and are also available upon request from the corresponding author.

## Author contributions

PZ and DVD performed conceptualization, data curation, formal analysis, supervision, and editing. CF, KM, NJC, AV, WL, JMP, JM, CYC, RAC, SB, CSFC, BC, PLM, CEH, SMS, DC, KR, and SP contributed data curation and formal analysis. ET and NH performed formal analysis. MD, RNG, YJK, and MC provided conceptualization and resources. GM participated in conceptualization, resources, and editing support. EEB performed conceptualization, resources, data curation, formal analysis, supervision, methodology, and editing. AEF performed data curation. First-authorship order was determined based on critical data generation.

## Supplementary Material

Supplemental data

Supporting data values

## Figures and Tables

**Table 1:**
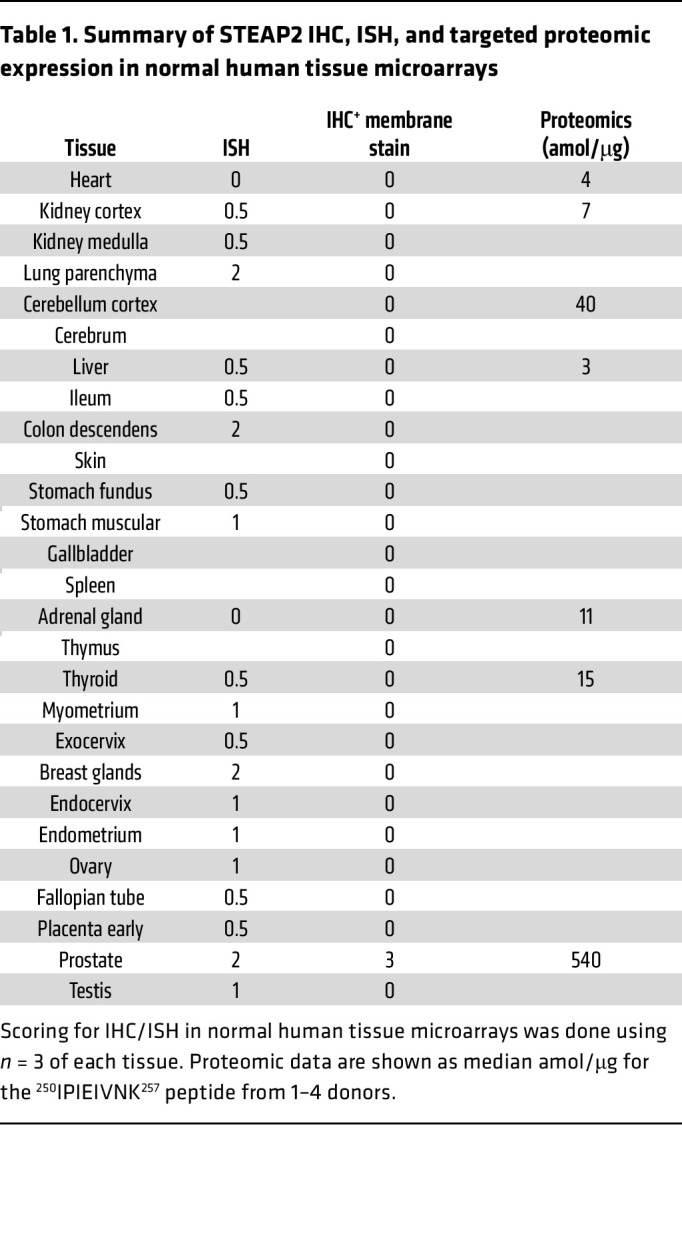
Summary of STEAP2 IHC, ISH, and targeted proteomic expression in normal human tissue microarrays

**Table 2:**
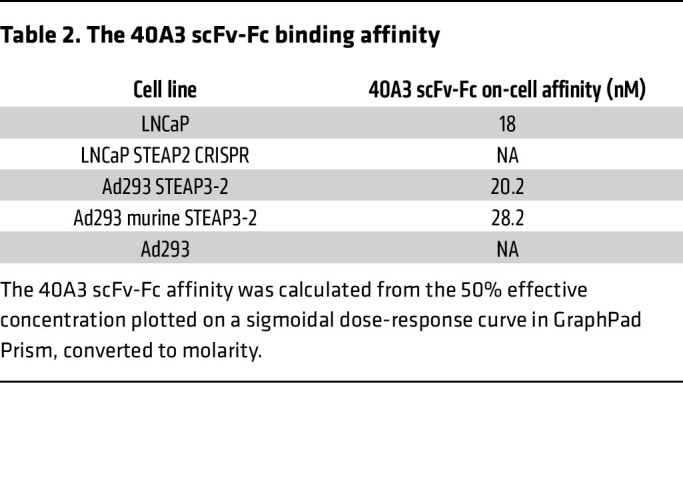
The 40A3 scFv-Fc binding affinity

## Version 1 11/15/2023

Electronic publication
