# Supplementary material for: Antitumor activity of AZD0754, a dnTGFβRII-armored, STEAP2-targeted CAR-T cell therapy, in prostate cancer
Source: J Clin Invest. 2023 Nov 15;133(22):e169655. doi: 10.1172/JCI169655 (PMC10645390; doi:10.1172/JCI169655)
Supplement: Supplemental data [file jci-133-169655-s055.pdf]

Antitumor activity of AZD0754, a dnTGFβRII-armored, STEAP2-targeted CAR-T cell therapy, in prostate cancer

Zanvit P, van Dyk D, Fazenbaker C, McGlinchey K, Luo W, Pezold J, Meekin J, Chang C, Carrasco RA, Breen S, Cheung CSF, Endlich-Frazier A, Clark B, Chu N, Vantellini A, Martin PL, Hoover C, Riley K, Sweet SM, Chain D, Kim YJ, Tu E, Harder N Phipps S, Damschroder M, Gilbreth R, Cobbold M, Moody G, Bosco EE

Supplemental Data

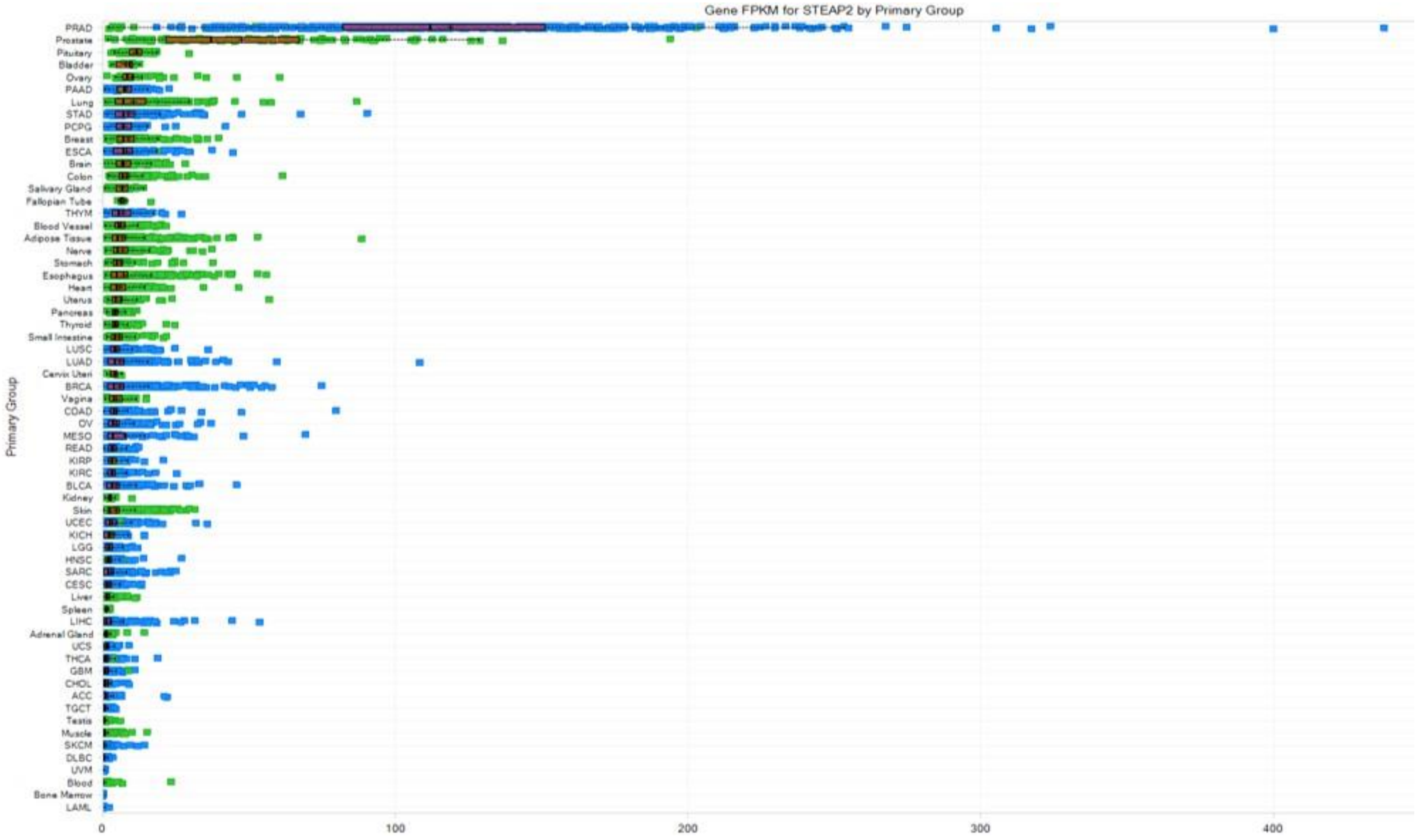

Supplemental Figure 1. The QIAGEN Omicssoft OncoLand genomic database was used to evaluate STEAP2 expression across normal human tissues and tumor types.

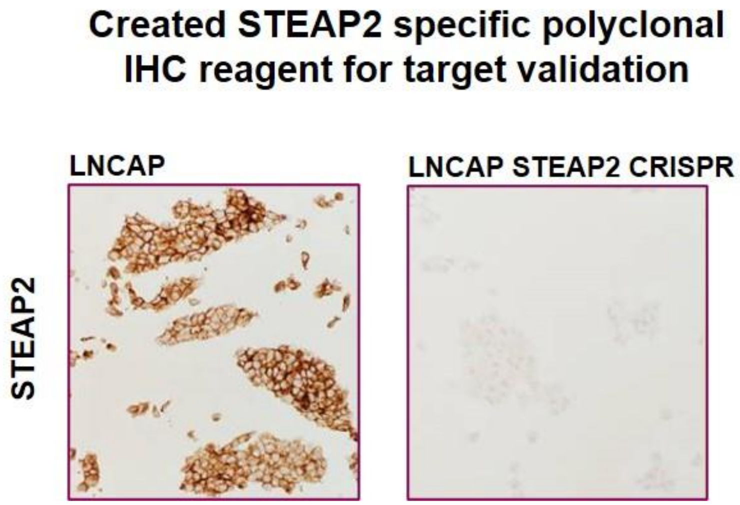

Supplemental Figure 2. Immunohistochemistry (IHC) was performed with anti-STEAP2 rabbit polyclonal antibody, which specifically identifies STEAP2 membrane staining in human prostate adenocarcinoma cell line LNCaP. No staining was detected in LNCaP cells with STEAP2 expression that had been eliminated using CRISPR knock-out.

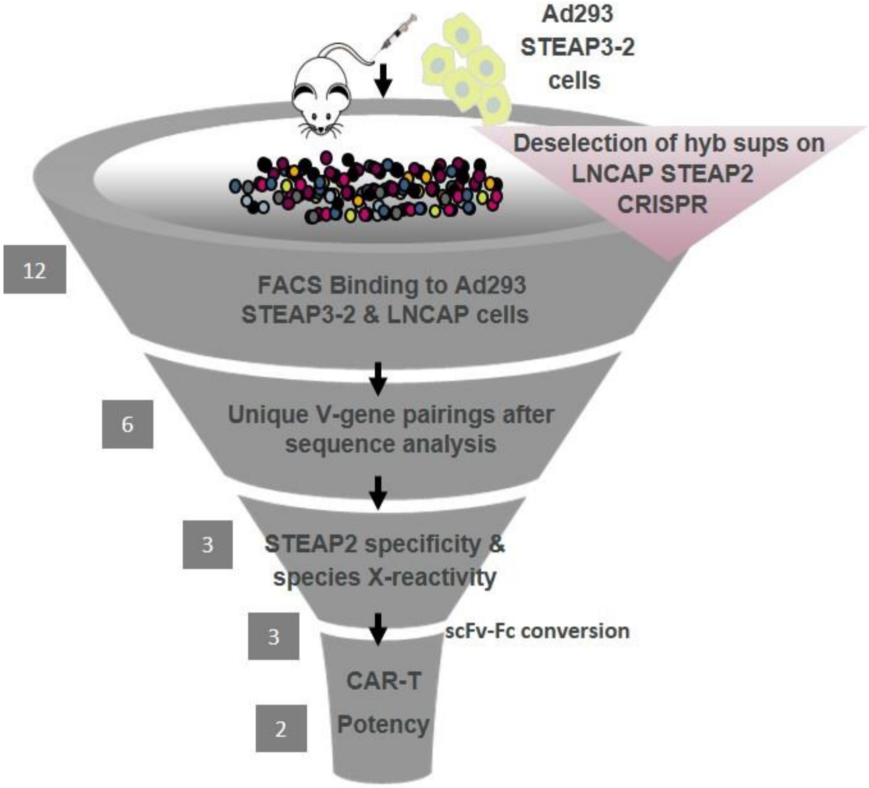

Supplemental Figure 3. Anti-STEAP2 antibody campaign performed in Del-1 mice immunized with Ad293 STEAP3-2 cells. Hybridoma supernatants were deselected in a binding assay with target-negative LNCaP STEAP2 CRISPR cells to enrich for STEAP2-specific binders, which were then screened by fluorescence-activated cell sorting (FACS) for binding to LNCaP and Ad293 STEAP3-2 cells but not LNCaP STEAP2 CRISPR or Ad293 parental cells. Clones were further triaged based on unique sequence analysis, cross-reactivity to murine STEAP2, and lack of binding to cell lines expressing STEAP1, 3, and 4 by FACS. The most promising immunoglobulin Gs were then converted to scFv-Fc format for evaluation of chimeric antigen receptor–T-cell (CAR-T) potency and lead selection.

>NP_694544.2 metalloreductase STEAP2 isoform a [Homo sapiens]

MESISMMGSPKSLSETFLPNGINGIKDARKVTVGVIGSGDFAKSLTIRLIRCGYHVVIGSRNPKFASEFFPHVVDVTHHEDALTKTNIIFVAIHREHYTSLWDLRHL
LVGKILIDVSNNMRINQYPESNAEYLASLFPDSLIVKGFNVVSAWALQLGPKDASRQVYICSNNIQARQQVIELARQLNFIPIDLGSLSSAREIENLPLRLFTLWRG
PVVVAISLATFFFL**YSFVRDVIHPYARNQQSDFYKIPIEIVNKT**LPIVAITLLSLVYLAGLLAAAYQLYYGTKYRRFPPWLETWLQCRKQLGLLSFFFAMVHVAYSL
CLPM**RRSERYLFLNMAYQQVHANIENSWNEEEVWRIE**MYISFGIMSLGLLSLLAVTSIPSVSNALNWREFSFIQSTLGYVALLISTFHVLIY**GWKRAFEEEYYRF**
YTPPNFVLALVLPSIVILGKIILFLPCISRKLKRIKKGWEKSQFLEEGMGGTIPHVSPERVTVM

>NP_878919.2 metalloreductase STEAP3 isoform a [Homo sapiens]

MSHQPAVATKMPEEMDKPLISLHLVDSDSSLAKVPDEAPKVGILGSGDFARSLATRLVGSGFKVVVGSRNPKRTARLFPSAAQVTFQEEAVSSPEVIFVAVFRE
HYSSLCSLSDQLAGKILVDVSNPTEQEHLQHRESNAEYLASLFPTCTVVKAFNVISAWTLQAGPRDGNRQVPICGDQPEAKRAVSEMALAMGFMPVDMGSL
ASAWEVEAMPLRLLPAWKVPTLLALGLFVCFYA**YNFVRDVLQPYVQESQNKFFKLPVSVVNTT**LPCVAYVLLSLVYLPGVLAAALQLRRGTKYQRFPDWLDH
WLQHRKQIGLLSFFCAALHALYSFCLPL**RRAHRYDLVNLAVKQVLANKSHLWVEEEVWRME**IYLSLGVLALGTLSLLAVTSLPSIANSLNWREFSFVQSSLGFV
ALVLSTLHTLTY**GWTRAFEESRYKFYLPPT**FTLTLLVPCVVILAKALFLLPCISRRLARIRRGWERESTIKFTLPTDHALAEKTSHV>

STEAP3-2 chimera [Homo sapiens]

MSHQPAVATKMPEEMDKPLISLHLVDSDSSLAKVPDEAPKVGILGSGDFARSLATRLVGSGFKVVVGSRNPKRTARLFPSAAQVTFQEEAVSSPEVIFVAVFRE
HYSSLCSLSDQLAGKILVDVSNPTEQEHLQHRESNAEYLASLFPTCTVVKAFNVISAWTLQAGPRDGNRQVPICGDQPEAKRAVSEMALAMGFMPVDMGSL
ASAWEVEAMPLRLLPAWKVPTLLALGLFVCFYA**YSFVRDVIHPYARNQQSDFYKIPIEIVNKT**LPCVAYVLLSLVYLPGVLAAALQLRRGTKYQRFPDWLDHW
LQHRKQIGLLSFFCAALHALYSFCLPL**RRSERYLFLNMAYQQVHANIENSWNEEEVWRIE**IYLSLGVLALGTLSLLAVTSLPSIANSLNWREFSFVQSSLGFVALV
LSTLHTLTY**GWKRAFEEEYYRFYTPPN**FTLTLLVPCVVILAKALFLLPCISRRLARIRRGWERESTIKFTLPTDHALAEKTSHV

Supplemental Figure 4. Amino acid sequences of human STEAP2 and STEAP3 used to generate the STEAP3-2 chimeric cell surface protein.

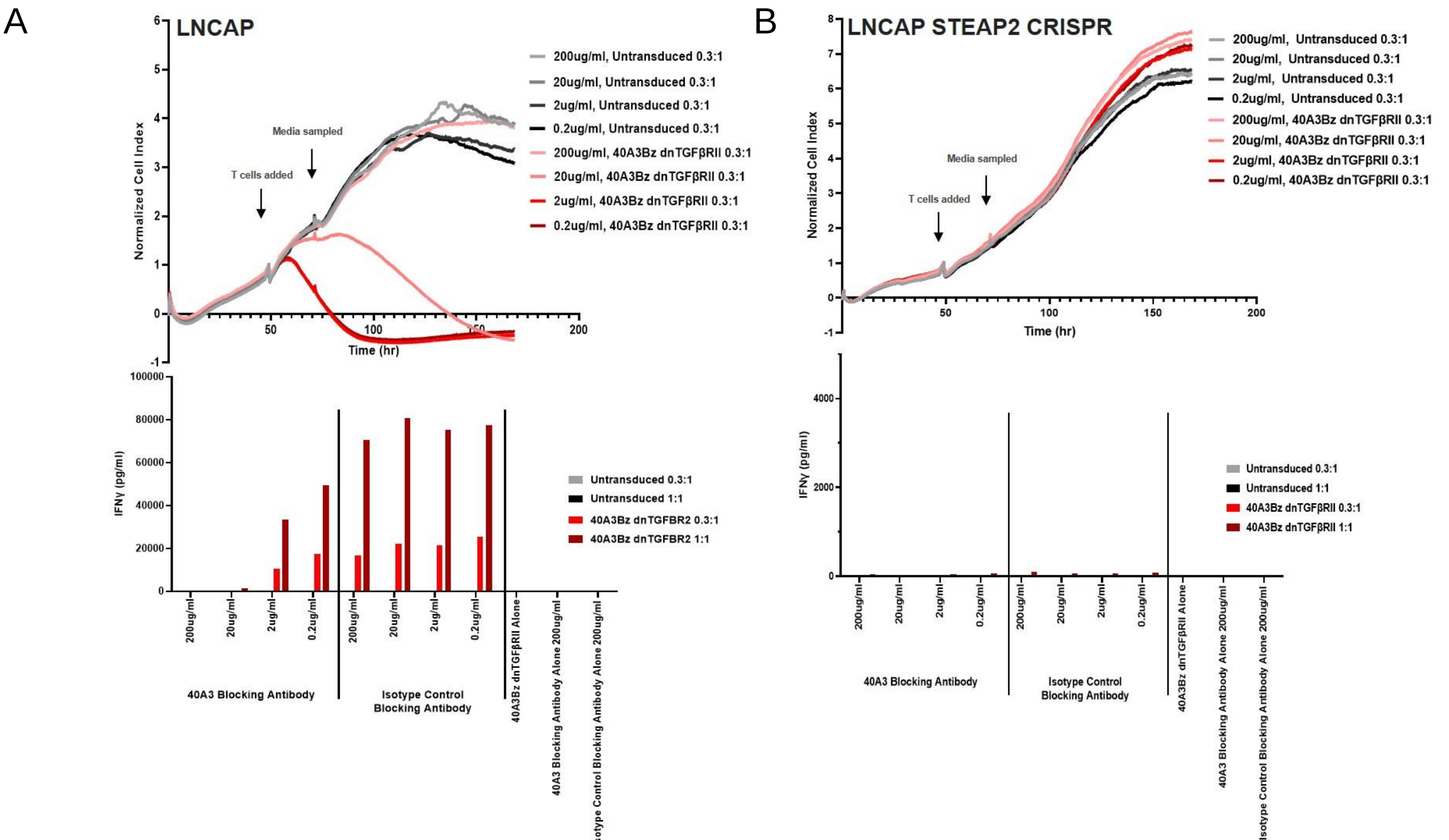

Supplemental Figure 5. Co-cultures were set up with LNCaP (**A**) and LNCaP STEAP2 CRISPR (**B**) tumor cell lines and 40A3Bz dnTGFβRII CAR-Ts at effector-to-target (E:T) ratios of 0.3:1 or 1:1. In addition, anti-STEAP2 or isotype blocking antibodies were administered to the culture (0.2, 2, 20, or 200 μg/mL). Tumor cell line growth was monitored by the xCELLigence system (Acea Biosciences) for 170 hours, and supernatants were sampled and evaluated for the presence of cytokines (interferon gamma [IFNγ], tumor necrosis factor alpha, and interleukin 2) with the Meso Scale Discovery (MSD) electrochemiluminescence (ECL) assay.

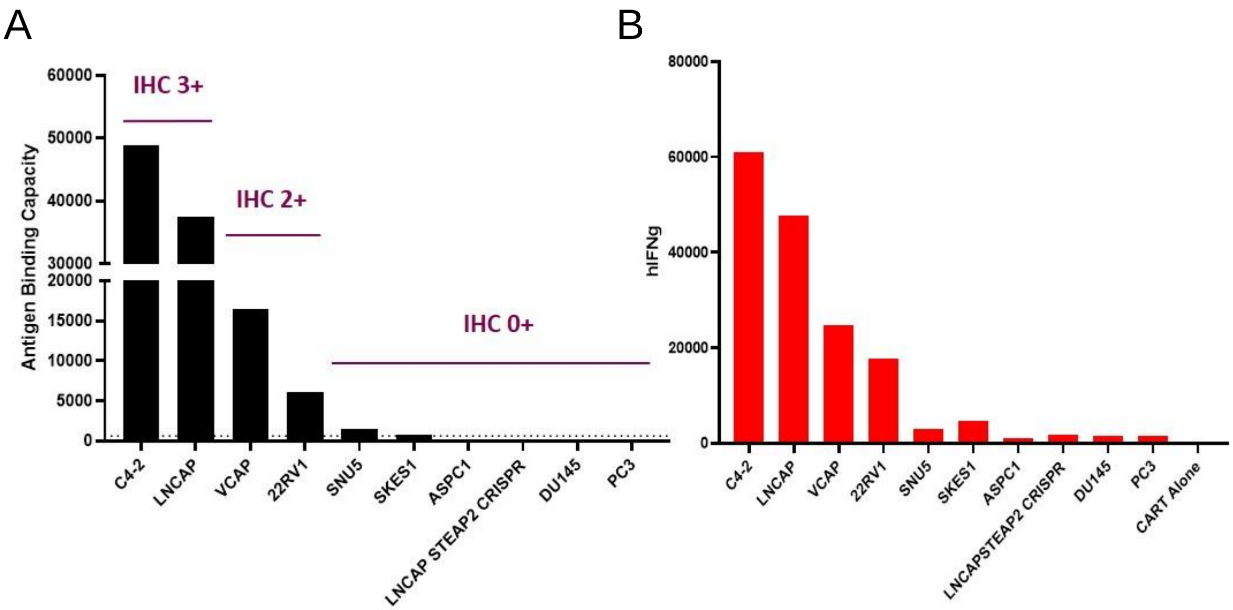

Supplemental Figure 6. A range of tumor cell lines was profiled by FACS, using an anti-STEAP2 antibody−Alexa Fluor 647 conjugate for antibody binding capacity and the Quantum Simply Cellular kit (Bangs Laboratories). **A,** STEAP2 cell surface IHC was performed on the same cell lines and cells were quantified. These cell lines were included in 40A3Bz dnTGFβRII CAR-T co-culture assays at an E:T ratio of 1:1. **B,** The medium was sampled at 24 hours to analyze the levels of IFNγ release from the CAR-Ts.

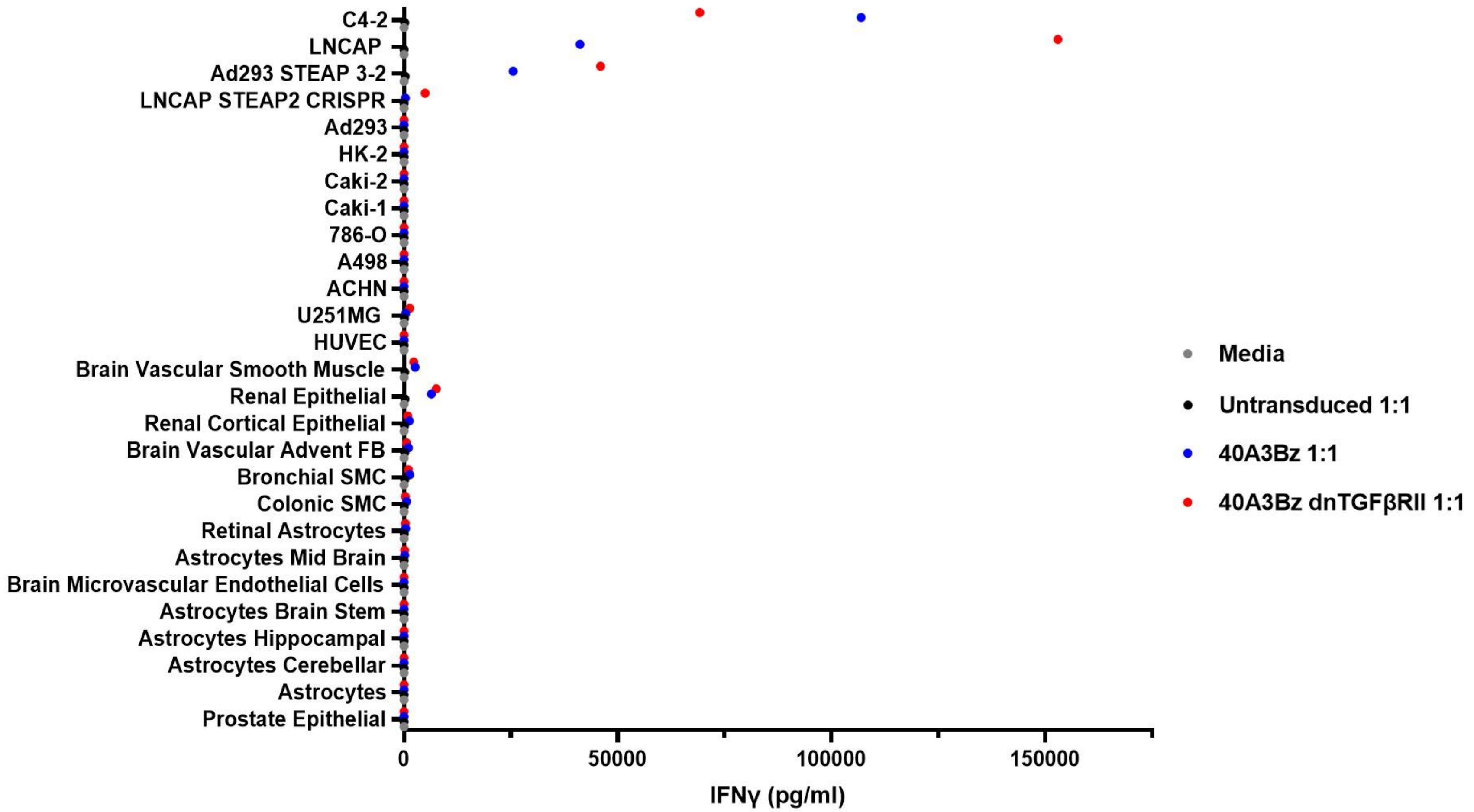

Supplemental Figure 7. Positive (C4-2, LNCaP, Ad293-STEAP3-2) and negative (LNCaP STEAP2 CRISPR, Ad293) STEAP2 control cell lines, as well as a variety of cancer cell lines and normal human primary cells, were co-cultured with 40A3Bz cells and 40A3Bz dnTGFβRII STEAP2 CAR-Ts at an E:T ratio of 1:1. Supernatants were sampled at 24 hours to profile for IFNγ with the MSD ECL assay.

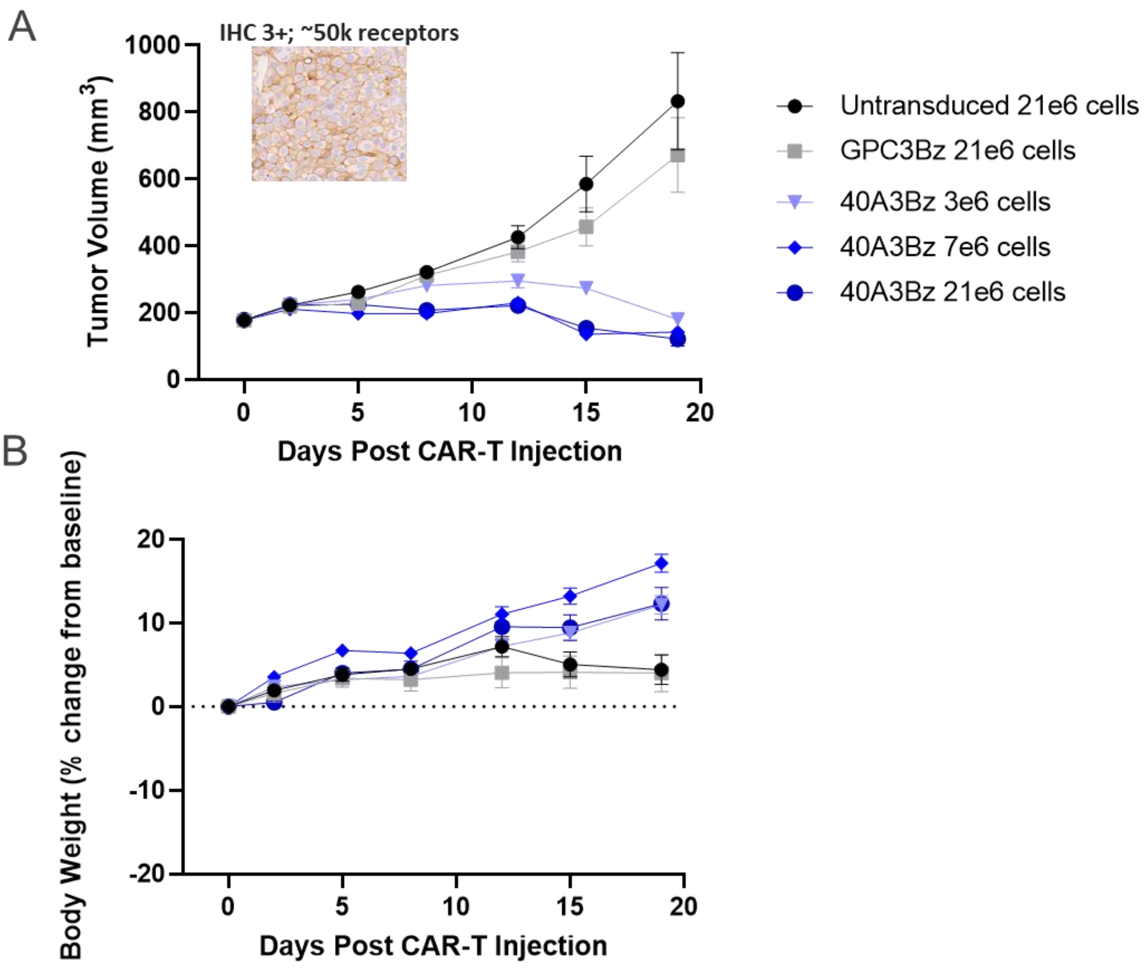

Supplemental Figure 8. 40A3Bz elicited in vivo tumor control without clinical signs of toxicity. The tolerability of unarmored 40A3Bz CAR-Ts was evaluated in NSG mice engrafted with the C4-2 prostate cancer cell line at three dose levels (3e6, 7e6, and 21e6 CAR-positive cells). Tumors were implanted 1:1 in Cultrex basement membrane extract (R&D Systems) in the flank, and tumor volume was measured for 35 days after implantation. Mice were randomized when tumors reached 175 mm^3, and T cells were administered once via tail vein injection (n=10). Tumor volume (**A**) and body weight (**B**) of the mice were measured up to day 28 after xenograft. Six mice from each group were sacrificed at day 10 after CAR-T infusion, and heart, liver, kidney, spleen, prostate, skin, and inflated lungs were harvested for IHC evaluation of CD3 infiltration and hematoxylin-eosin staining.

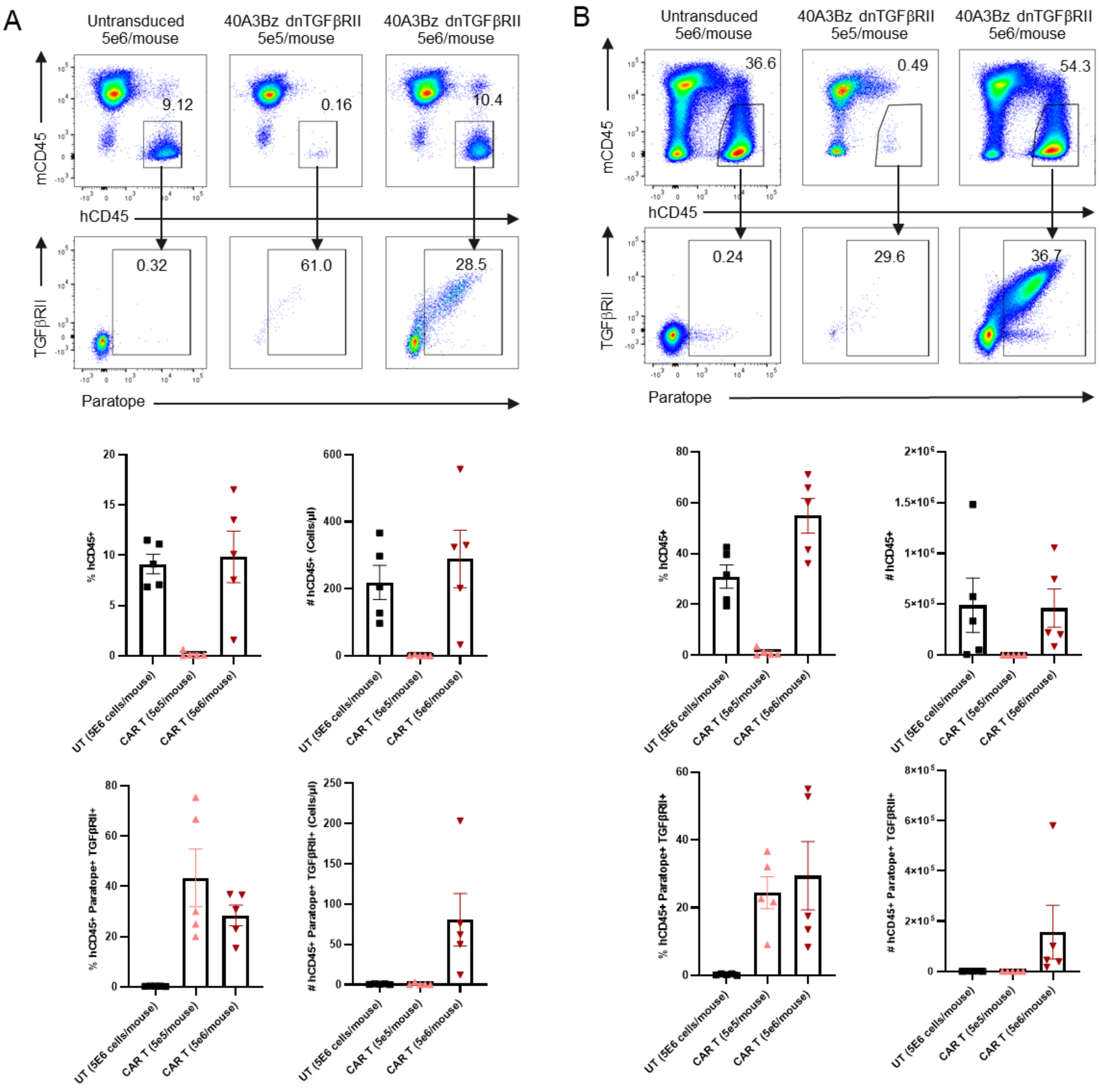

Supplemental Figure 9. (**A**) Blood from NSG MHC 1/2 DKO mice injected with Untransduced T cells (5E6 cells/mouse, n=5), 40A3Bz dnTGFβRII CAR-T (5E5 cells/mouse, n=5) or 40A3Bz dnTGFβRII CAR-T (5E6 cells/mouse, n=5) and implanted with LuCaP86.2 PDX tumors was collected on day 42. Percentage and total number (Cells/µl) of hCD45$^+$ as well as hCD45$^+$ Paratope$^+$ TGFβRII$^+$ cells were determined by flow cytometry. (**B**) Spleens from mice described in **A** were collected at day 42. Percentage and total number of hCD45$^+$ as well as hCD45$^+$ Paratope$^+$ TGFβRII$^+$ cells were determined by flow cytometry. Representative FACS data provided above with individual data provided from an n=5 animals below. All error is shown as +/- SEM.
